# Hypertrophic scar contracture is mediated by the TRPC3 mechanical force transducer via NFkB activation

**DOI:** 10.1038/srep11620

**Published:** 2015-06-25

**Authors:** Hisako Ishise, Barrett Larson, Yutaka Hirata, Toshihiro Fujiwara, Soh Nishimoto, Tateki Kubo, Ken Matsuda, Shigeyuki Kanazawa, Yohei Sotsuka, Kazutoshi Fujita, Masao Kakibuchi, Kenichiro Kawai

**Affiliations:** 1 Department of Plastic Surgery, Hyogo College of Medicine, 1-1 Mukogawacho, Nishinomiya, Hyogo 663-8501, Japan; 2 Department of Anesthesiology, Pain & Perioperative Medicine Stanford University School of Medicine, 300 Pasteur Dr, Stanford, CA 94305 USA; 3 Department of Physiology, Hyogo College of Medicine, 1-1 Mukogawacho, Nishinomiya, Hyogo 663-8501, Japan; 4 Department of Plastic Surgery, Osaka University Graduate School of Medicine, 2-2-C11, Yamadaoka, Suita, Osaka 565-0871, Japan

## Abstract

Wound healing process is a complex and highly orchestrated process that ultimately results in the formation of scar tissue. Hypertrophic scar contracture is considered to be a pathologic and exaggerated wound healing response that is known to be triggered by repetitive mechanical forces. We now show that Transient Receptor Potential (TRP) C3 regulates the expression of fibronectin, a key regulatory molecule involved in the wound healing process, in response to mechanical strain via the NFkB pathway. TRPC3 is highly expressed in human hypertrophic scar tissue and mechanical stimuli are known to upregulate TRPC3 expression in human skin fibroblasts *in vitro*. TRPC3 overexpressing fibroblasts subjected to repetitive stretching forces showed robust expression levels of fibronectin. Furthermore, mechanical stretching of TRPC3 overexpressing fibroblasts induced the activation of nuclear factor-kappa B (NFκB), a regulator fibronectin expression, which was able to be attenuated by pharmacologic blockade of either TRPC3 or NFκB. Finally, transplantation of TRPC3 overexpressing fibroblasts into mice promoted wound contraction and increased fibronectin levels *in vivo*. These observations demonstrate that mechanical stretching drives fibronectin expression via the TRPC3-NFkB axis, leading to intractable wound contracture. This model explains how mechanical strain on cutaneous wounds might contribute to pathologic scarring.

Wound contraction is an important component of the cutaneous wound healing process^1^,^2^,^3^. Failure to contract can result in a variety of pathologic skin conditions, such as chronic ulceration. However, excessive wound contraction can result in scar contracture with fibrosis. Pathologic scarring is the result of an exaggerated wound healing response characterized by scar contracture and over-deposition of extracellular matrix (ECM)^1^,^4^. Hypertrophic contractile fibrosis tends to occur in areas undergoing repetitive mechanical stretch stimulations, such as at the joints and mouth^5^,^6^,^7^. However, the link between mechanical stretching and cutaneous scar formation/contracture is still largely unknown.

Fibronectin is a high-molecular weight glycoprotein that is produced by various different cell types and assembled into the ECM^8^. During cutaneous wound healing, fibronectin is robustly secreted from fibroblasts. Fibronectin not only serves as scaffolding support, but also functions as the endogenous ligand that is responsible for regulating several wound healing responses^9^,^10^. Fibronectin induces cell differentiation, migration, coagulation and formation of ECM, which ultimately serves to promote wound contraction^10^,^11^,^12^,^13^,^14^.

The expression of fibronectin is highly regulated and known to respond to various signals, such as TGF-β^15^,^16^,^17^. Multiple signaling molecules interact with specific regions of the fibronectin promoter and regulate expression of the gene^18^,^19^,^20^. Among the transcription factors known to interact with the fibronectin promoter, the well-studied protein, nuclear factor-kappa B (NF-κB) has been shown to regulate fibronectin expression by binding to the kappa-B binding motif (GGGACTTTCC)^21^,^22^. NF-κB is involved in the control of a large number of biological processes, such as inflammatory and immune responses, cancer growth^23^,^24^ and fibrotic diseases^25^,^26^. Given NFκB’s known influence on fibronectin expression, we postulated that the NFκB signaling pathway may contribute to the process of cutaneous wound contraction.

One of the factors known to activate NFkB is intracellular calcium^26^,^27^, the influx of which is mediated by calcium channels. Recently, an ion channel group called Transient-Receptor-Potential (TRP) has been identified as a possible mechanical force transducer^28^,^29^,^30^,^31^,^32^. TRP channels are widely expressed in many different tissues and cell types, where they are involved in diverse physiological processes, such as sensation of different stimuli or ion homeostasis. However, to date, the role of TRPC channels in wound contraction has not been fully elucidated. Although it is well established that TRPC exerts its function through the Calcineurin/NFAT pathway in cardiomyocytes^29^,^31^,^33^,^34^, the possible relationship between TRPC and NFκB has not previously been investigated.

In the present study, we sought to investigate the mechanism by which repetitive mechanical stretching causes wound contraction. Our study focused on the mechanical force transducer TRPC3, and its role in regulating fibronectin expression via the NFκB pathway.

## Results

### Cyclic Stretching on human fibroblasts increases expression of fibronectin

**Figure 1:**
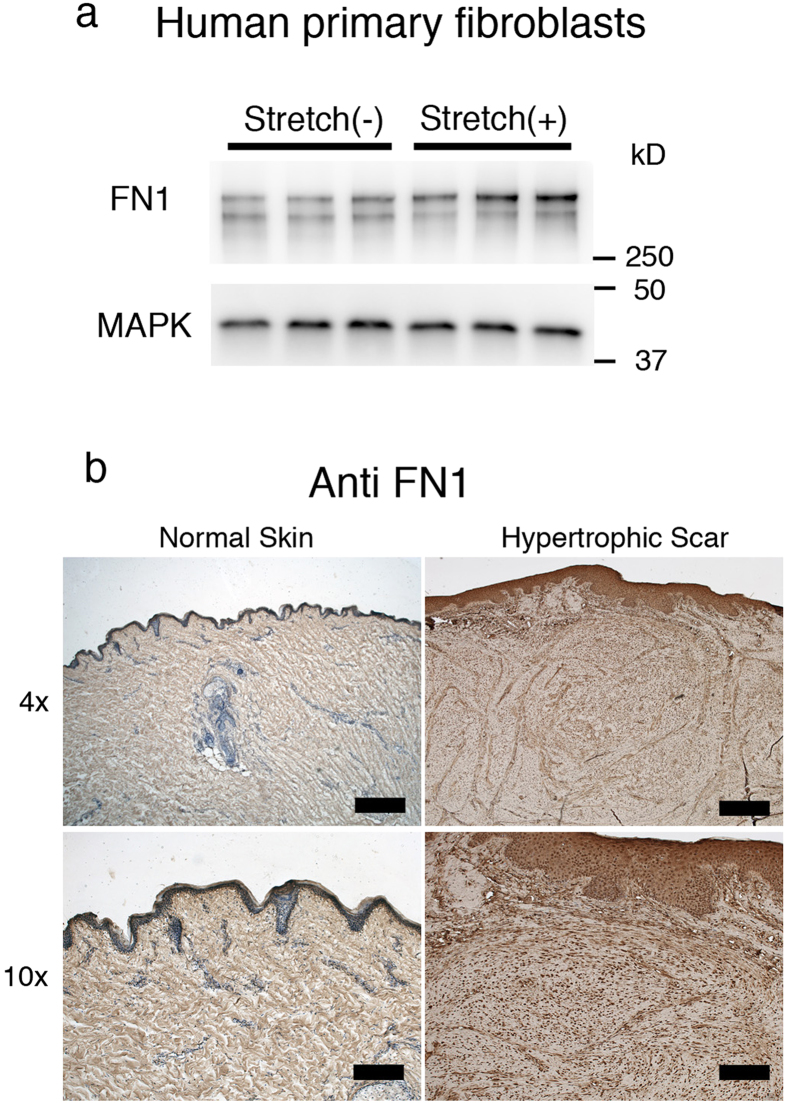
Expression of Fibronectin in human hypertrophic scar tissue. Fibronectin is one of the key molecules in hypertrophic scar contracture. We investigated whether mechanical stretching increases the expression of Fibronectin. **a**; Western Blot analysis with anti-Fibronectin antibody demonstrated that the expression of Fibronectin protein in human primary fibroblasts was increased when they were subjected to 24 hours of repetitive stretching. There were 3 samples per group. To improve the clarity and the conciseness of the data, the blots were cropped. Uncropped, full-length blots are presented in Supplementary Figures S4 and 5. **b**; Immunohistochemical staining with anti-Fibronectin showed that Fibronectin was strongly stained around the cell cluster in human hypertrophic scar tissue whereas the fibrous connective structure was homogenously stained in normal skin tissue. Scale bars represent 500 μm in 4× images and 200 μm in 10× images.

Hypertrophic scar contracture tends to occur in areas undergoing repetitive mechanical stretching, where increased fibronectin expression is observed. In order to determine if stretching forces may influence the expression level of fibronectin, human primary fibroblasts were stretched and the expression level of fibronectin was measured using Western Blotting. Our results demonstrate that human fibroblasts produce more fibronectin when exposed to repetitive mechanical stretching (Fig. 1a). In samples of human hypertrophic scar tissue, which had previously been exposed to repetitive stretching, immunohistochemical analysis revealed that the distribution of fibronectin was organized with the structure of the lesion. However, in samples of normal skin tissue, there was homogenous staining of fibronectin (Fig. 1b). The immunostaining pattern was consistent with a previous study by Kischer *et al*.^35^. These results suggest that repetitive stretching may induce fibronectin expression in fibroblasts and result in scar contracture.

### TRPC3 expression in stretched human scar tissue is increased

**Figure 2:**
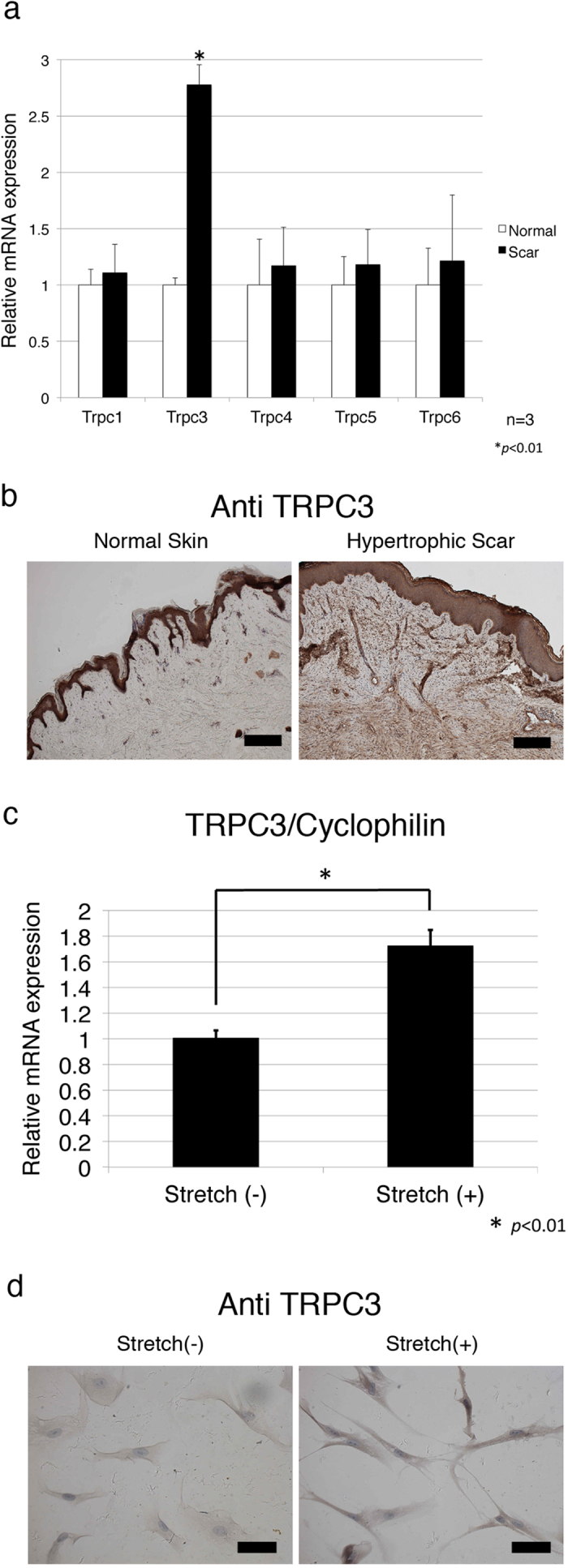
Expression of TRPC3 channel in human hypertrophic scar tissue. In order to investigate the expression levels of TRPC3 in human hypertrophic scar tissue, 3 surgical specimens were obtained from scar revision surgery and underwent qRT PCR and immunohistochemical analysis as described in the Materials and Methods. **a**; qRT-PCR showed human hypertrophic scar tissue had more TRPC3 expression compared to normal skin. Values were standardized against cyclophilin. Data represents means ± SD of 3 samples. *p < 0.05 by student t-test. **b**; TRPC3 channel protein in hypertrophic scar tissue was detected with anti-TRPC3 antigen. Representative photographs are shown. Immunohistochemistry demonstrated that human hypertrophic scar tissues were strongly stained with anti TRPC3 antibody compared to normal skin. Similar to Fibronectin staining,, TRPC3 was also strongly stained along with cell clusters in scar tissue. Scale bars represent 200 μm. **c**; mRNA expression of TRPC3 in human fibroblasts subjected to mechanical stretchng was measured using qRT-PCR as described in the Materials and Methods section. Stretched cells showed more TRPC3 expression compared to normal, unstretched fibroblasts. Data represents means ± SD of 3 samples. *p < 0.01 by student t-test **d**; Human primary fibroblasts were stretched (20%, 10 Hz) for 24 hours and then stained with anti TRPC3 antibody. Immunocytochemistry confirmed increased TRPC3 expression in human fibroblasts in response to mechanical stretching. Scale bar represents 20 μm.

To determine whether TRPC expression is related to hypertrophic scar contracture, we analyzed human hypertrophic scar samples obtained from surgical excisions. Human hypertrophic scar tissue, and normal skin obtained adjacent to the scar, were excised and the expression levels of TRPC transcripts were examined using quantitative real-time PCR. TRPC 1, 3, 4, 5 and 6 were detectable in the scar and the expression level of TRPC3 mRNA was significantly upregulated (Fig. 2a). The increased TRPC3 expression in human hypertrophic scar tissue was also seen with immunohistochemistry (Fig. 2b). Similar to the immunostaining pattern of fibronectin, TRPC3 expression was higher in contractile scar samples compared to normal skin samples. In human fibroblasts subjected to cyclic stretching, we found increased expression of TRPC3 mRNA (Fig. 2c). This result was confirmed via immunocytochemistry using an anti-TRPC3 antibody (Fig. 2d). Taken together, these results suggest that repetitive stretching up-regulates the expression of TRPC3 in fibroblasts, which may ultimately lead to scar contracture by upregulating fibronectin production.

### Repetitive stretching of TRPC3 overexpressing fibroblasts increases fibronectin production

**Figure 3:**
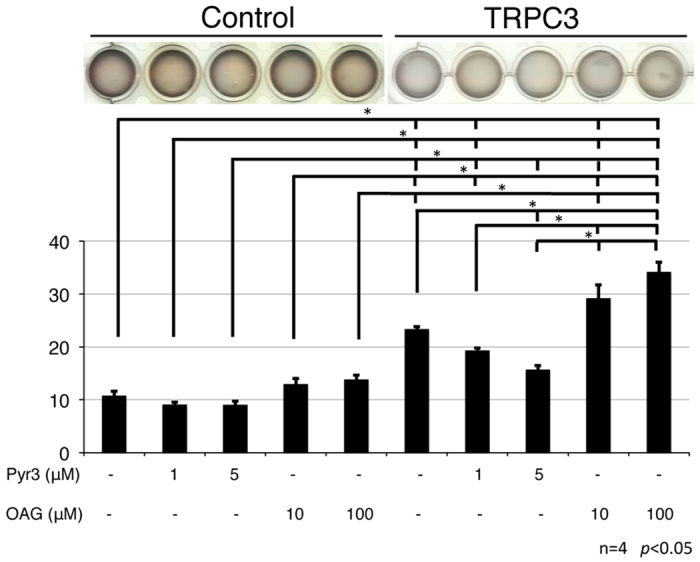
Fibroblast Populated Collagen Lattice with TRPC3 overexpressing fibroblasts appeared to have more contractile activity than a collagen lattice with control fibroblasts. The contractile activities of TRPC3 overexpressing fibroblasts were analyzed using a gel contraction assay. Each gel contained either 8 × 10^5^ TRPC3 overexpressing cells or empty vector transfected control cells and was incubated for 24 hours. After starvation, the gels were treated with either a TRPC3 agonist (OAG) or a TRPC3 inhibitor (Pyr3) and the contraction ratio was observed. Gels with TRPC3 overexpressing cells exhibited more contraction than control gels. This contraction effect was enhanced with OAG and attenuated by Pyr3. The data is expressed as the mean ± SD (n = 4). *p < 0.05 by Tukey-Kramer test.

In order to elucidate the mechanism by which TRPC3 regulates fibronectin production in response to mechanical stretching, we produced TRPC3 overexpressing fibroblasts. Our TRPC3 overexpressing cells had robust TRPC3 protein levels (supplemental Figure S1) and the protein function was confirmed using a calcium imaging technique (supplemental Figure S2). BrdU uptake analysis showed that the proliferative activity of these cells was not altered compared to both empty-vector transfected cells and normal NIH3T3 cells (supplemental Figure S3). However, a Fibroblast Populated Collagen Lattice (FPCL) analysis showed that TRPC3 overexpressing cells had more contractile activity than control cells. The contraction of gels containing TRPC3 overexpressing fibroblasts was accelerated by the TRPC agonist OAG, and attenuated by the TRPC3 specific inhibitor Pyr3 (Fig. 3). This finding further suggests that the TRPC3 channel mediates wound contracture by altering the contractile activity of fibroblasts.

**Figure 4:**
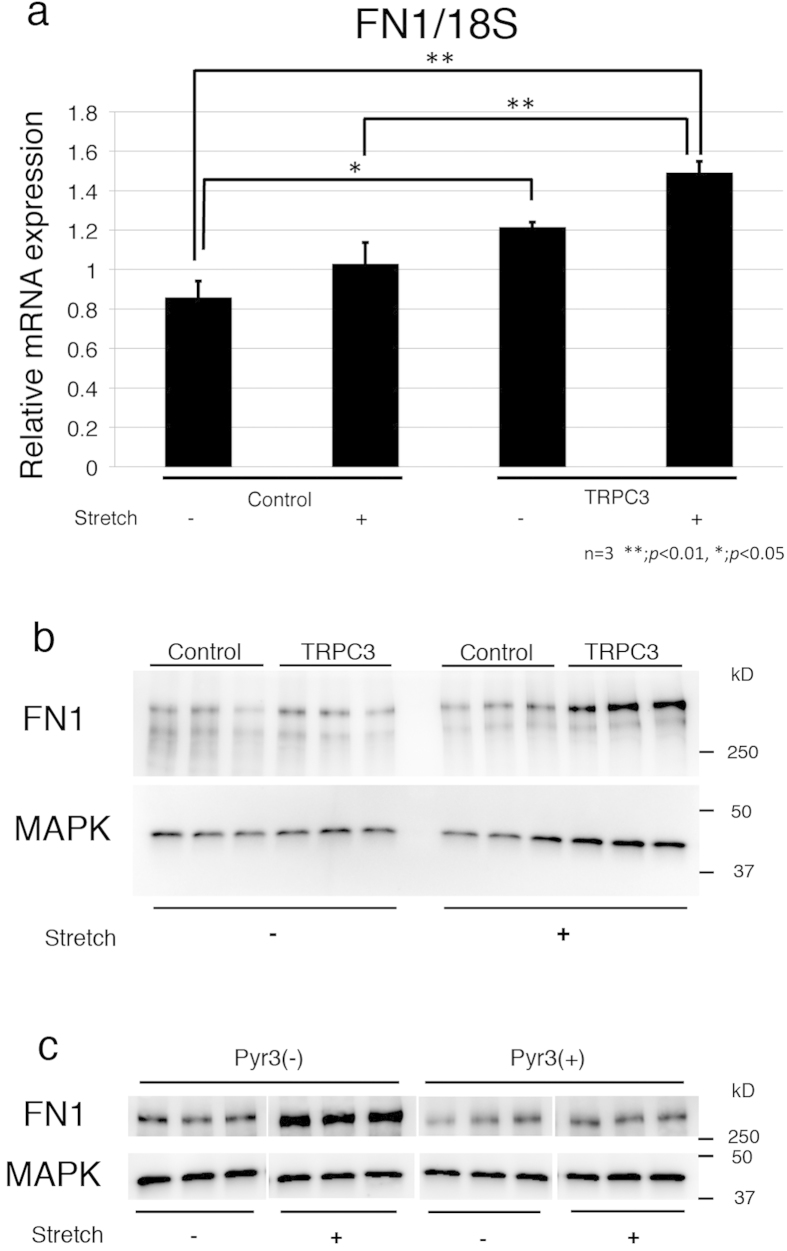
Fibronectin production was increased in TRPC3 overexpressing fibroblasts when they were subjected to repetitive stretching. TRPC3 overexpressing fibroblasts and control cells were stretched for 24 hours and the expression levels of Fibronectin were assessed using qRT PCR and Western Blot analysis. The blots presented here were cropped to improve clarity. Uncropped, full-length blots are presented in Supplementary Figures S6–10. **a**; qRT PCR demonstrated that the gene expression level of Fibronectin was significantly increased after 24 hours of stretching in TRPC3 overexpressing cells compared to control cells. Data represents means ± SD of 3 samples. *p < 0.05 by Tukey-Kramer test. **b**; Immunoblotting data demonstrated that the production of Fibronectin was markedly enhanced when TRPC3 overexpressing cells were stretched for 24 hours (n = 3). **c**; Increased Fibronectin production in TRPC3 overexpressing cells subjected to 24 hours stretching was attenuated by the addition of Pyr3 (n = 3).

Because fibronectin is known to promote wound contraction, its expression levels were examined in TRPC3 overexpressing fibroblasts exposed to repetitive stetching. Inducing a 20% stretch at a frequency of 10 times per minute for 24 hours increased mRNA expression of fibronectin in TRPC3 overexpressing cells (Fig. 4a). The increased production of fibronectin in TRPC3 overexpressing cells in response to the stretching was confirmed with Western Blot analysis using anti-fibronectin antibody (Fig. 4b). The TPRC3 specific inhibitor Pyr3 attenuated the increased production of fibronectin that occurred in response to stretching (Fig. 4c). These results demonstrate that the TRPC3 channel transduces mechanical stimuli into a biologic cell-signaling cascade that leads to the increased production of fibronectin.

### Repetitive stretching of TRPC3 overexpressing fibroblasts activates NFκB

**Figure 5:**
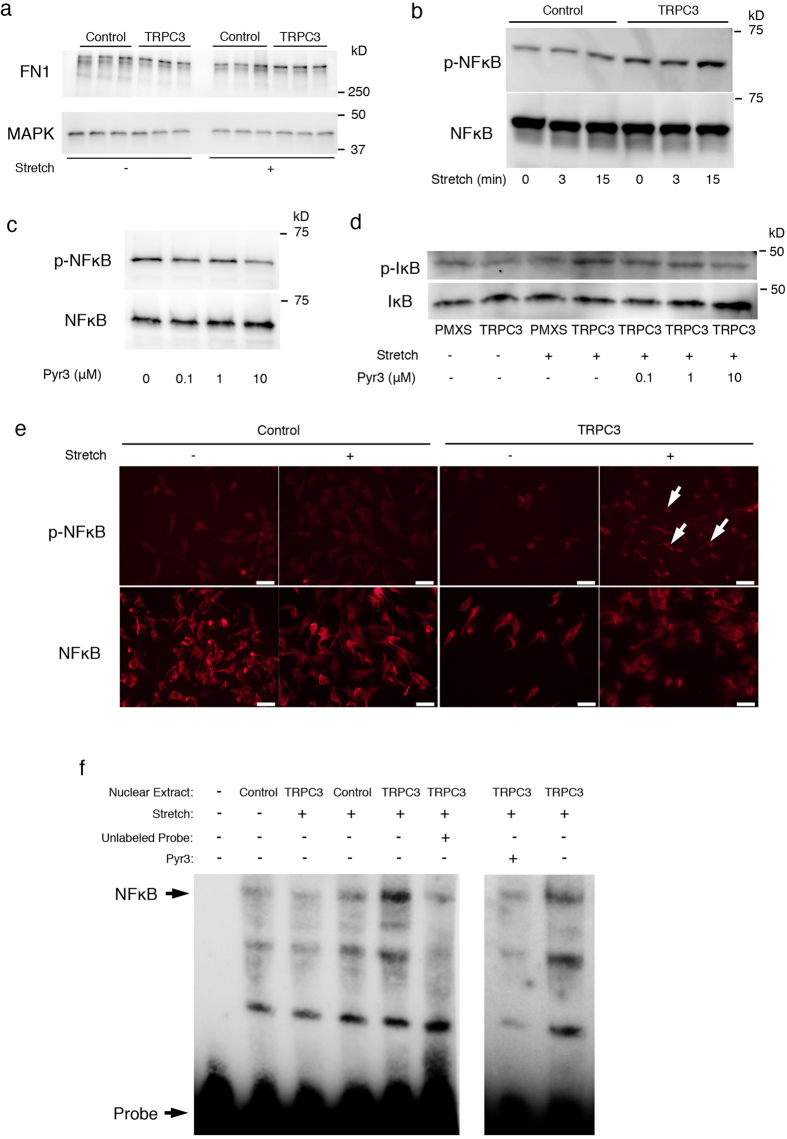
TRPC3 mediates cyclic stretching forces via NFκB activation in fibroblasts. To elucidate the mechanism by which Fibronectin expression is increased in TRPC3 overexpressing cells in response to mechanical stretching, the activity of transcriptional factor NFκB was assessed. To clarify the data, the blots were cropped. Uncropped, full-length blots for Western Blot and EMSA are presented in Supplementary Figures S11–20. **a**; In order to determine whether NFκB is involved in the regulation of Fibronectin production, TRPC3 overexpressing cells and control cells were stretched (20%, 10 Hz) with/without an NFκB inhibitor (Wedelolactone) for 24 hours. Western blot analysis demonstrated that the increased expression of Fibronectin in TRPC3 overexpressing cells in response to mechanical stretching was blocked by Wedelolactone. **b**; The phosphorylation of NFκB in TRPC3 overexpressing cells was upregulated in a time dependent manner compared to control cells **c**; The phosphorylation of NFκB in response to stretching stimuli was attenuated by the TRPC3 specific inhibitor, Pyr3. **d**; The phosphorylation pattern of IκB, which is a regulator of NFκB activation, was assessed. TPC3 overexpressing fibroblasts showed more activation of IκB in response to mechanical stretching compared to control fibroblasts. The activation of IκB was attenuated by Pyr3 in dose dependent manner. **e**; Translocation of phosphorylated NFκB was evident in TRPC3 overexpressing fibroblasts stretched for 15 minutes. Arrows indicate translocated phosphorylated NFκB. Scale bars for each images = 50 μm. **f**; TRPC3 overexpressing fibroblasts and control fibroblasts were stretched for 15 minutes and the nuclear proteins were extracted. A gel shift assay was then performed, as described in the Materials and Methods section. Gel shift was most apparent in stretched TRPC3 overexpressing fibroblasts, which was attenuated by the addition of Pyr3.

It has previously been reported that the fibronectin promoter has a kappa B binding motif (GGGACTTTCC) and the NFκB pathway regulates the expression of fibronectin^21^,^22^. In our study, the NFkB inhibitor Wedelolactone was able to attenuate fibronectin production in TRPC3 overexpressing fibroblasts that were repetitively stretched (Fig. 5a). This result indicates that fibronectin expression is regulated by NFκB. We also investigated whether NFκB is activated in TRPC3 overexpressing fibroblasts in response to repetitive stretching. The cell lysate from stretched fibroblasts was analyzed using Western Blot analysis. In TRPC3 overexpressing fibroblasts, repetitive stretching caused phosphorylation of NFκB in a time dependent manner (Fig. 5b). To test whether the stretch-induced activation of NFκB is mediated by TRPC3, we added the TRPC3 specific inhibitor Pyr3 to the growth medium during cell stretching and found that Pyr3 attenuated the phosophorylation of NFκB in repetitively-stretched TRPC3 overexpressing cells (Fig. 5c). We also investigated whether repetitive stretching of TRPC3 overexpressing cells induces IκB phosphorylation, which is the crucial step required for NFκB activation. Figure 5d shows that IκB phosphorylation was higher in stretched TRPC3 overexpressing fibroblasts compared to control or unstretched TRPC3 overexpressing fibroblasts. Of note, IkB phosphorylation was also attenuated by the addition of Pyr3. Immunocytochemical analysis using an anti-phospho- NFκB antibody demonstrated that the translocation of phosphorylated NFκB into nuclei was enhanced in TRPC3 overexpressing fibroblasts that were subjected to stretching (Fig. 5e). Finally, NFκB activity in TRPC3 overexpressing fibroblasts was examined with an EMSA assay. Nuclear extracts from either stretched, unstretched control, or TRPC3 overexpressing fibroblasts were reacted with a kappa-B motif oligonucleotide, followed by separation via electrophoresis and ultimately visualized by transblotting. As shown in Fig. 5f, gel shift was induced in stretched TRPC3 overexpressing fibroblasts and the shift was competed by addition of unlabeled oligonucleotide representing kappa-B binding motif, confirming the activity of NFκB. Adding the inhibitor Pyr3 also diminished the shift. These results strongly suggest that the enhanced activity of NFκB in repetitively stretched TRPC3 overexpressing fibroblasts is mediated by TRPC3.

### Repetitive stretching in TRPC3 overexpressing fibroblasts induces calcium influx

**Figure 6:**
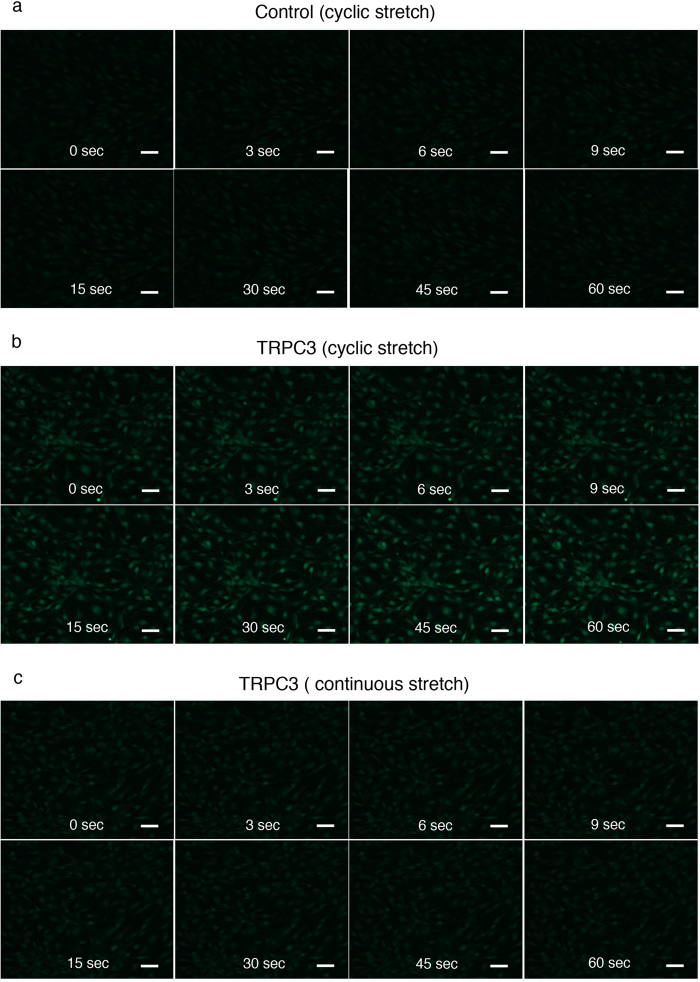
Real-time calcium imaging of TRPC3 overexpressing fibroblasts and control fibroblasts during mechanical stretching. Live imaging of calcium influx in fibroblasts was obtained as described in the Materials and Methods section. Scale bars for each images = 50 μm. **a**; Control fibroblasts showed a weak increase in fluorescence upon repetitive mechanical stretching. **b**; TRPC3 overexpressing fibroblasts exhibited increased fluorescence upon repetitive mechanical stretching. The increased fluorescence continued throughout the period in which they were stretched. **c**; When TRPC3 overexpressing fibroblasts were stretched once and then held in a stationary position, the fluorescence decreased over time.

In order to determine whether repetitive stretching of TRPC3 overexpressing cells directly changes calcium influx, we employed a calcium imaging technique using the calcium indicator Fluo4-AM. TRPC3 overexpressing fibroblasts underwent rapid calcium influx in response to repetitive stretching and the fluorescence intensified with each stretch when compared to control cells (Fig. 6a,b. Supplemental video 1, 2, 3, 4). In contrast, TRPC3 overexpressing fibroblasts that were *continuously* stretched experienced decreasing fluorescence over time (Fig. 6c. Supplemental video 1, 2, 3, 4).

### Transplantation of TRPC3 overexpressing fibroblasts into mice increases wound contraction

**Figure 7:**
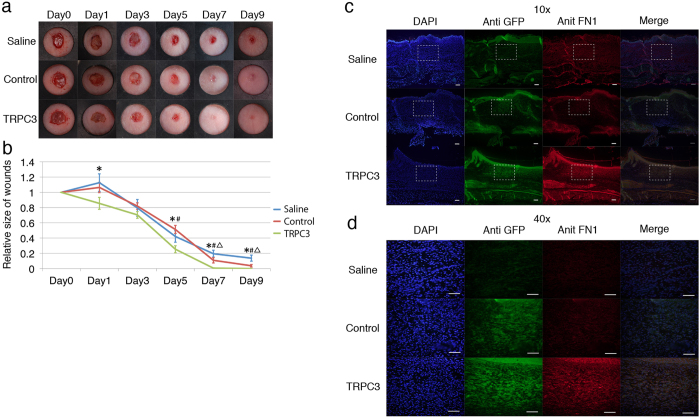
Wound contraction was enhanced by the transplantation of TRPC3 overexpressing fibroblasts in mice. To assess the effect of TRPC3 overexpressing fibroblasts on wound healing *in vivo*, TRPC3 overexpressing fibroblasts, empty-vector transfected control fibroblasts or the same amount of saline were injected into the dorsum of nude mice. 10 days post-transplantation, 6 mm wounds were created and the size of the wounds were subsequently measured on Days 1, 3, 5, 7 and 9. The wound tissue was ultimately harvested at Day 9 post-wounding and immunochemistry with anti-TRPC3 and Fibronectin was performed. **a**; Representative photographs of cutaneous wounds. Differential healing was observed at all time points checked (n = 6 for each time point). **b**; Quantification of the wound area from Days 0–9. The data is expressed as the mean ± SD. n = 6 per group. *p < 0.05 TRPC3 versus Saline. *p* < 0.05 TRPC3 versus PMXS. *p* < 0.05 PMXS versus Saline by Tukey-Kramer test. **c**; Immunohistochemistry representing the expression of Fibronectin in the wound tissue after transplantation of TRPC3 overexpressing cells, control cells, and normal saline. Sections were taken from the center of wounds and stained with anti TRPC3 and Fibronectin antibodies. Wounds transplanted with TRPC3 overexpressing cells express Fibronectin more than control wounds. **d**; Magnified images of Fig. 7C. Fibronectin staining was more evident around TRPC3 overexpressing cells.

The effect of TRPC3 overexpressing fibroblasts on wound contraction was investigated *in vivo* on a 6 mm full-thickness excisional mouse skin wound model. Mice received a subcutaneous injection of control empty vector transfected fibroblasts, TRPC3 overexpressing fibroblasts, or the same volume of saline 10 days prior to injury. At all time points post-injury, the rate of wound closure was found to be significantly faster in the mice treated with TRPC3 overexpressing cells (Fig. 7a,b). Wounds were stained with anti-fibronectin antibody to assess the amount of fibronectin deposition. Sections were taken from the center of the wounds. At Day 9, the wounds of mice treated with TRPC3 overexpressing fibroblasts had increased fibronectin deposition compared to controls (Fig. 7c,d).

## Discussion

Clinical observations suggest that hypertrophic scar contracture tends to occur in areas undergoing repetitive stretching, such as at the fingers, joints, or mouth. Although many scar treatments and prevention methods have been proposed, we are still searching for more effective strategies. Given that the detailed mechanisms underlying scar pathophysiology are largely unknown, our therapeutic options remain limited.

Contrary to hypertrophic scar contracture, insufficient contraction of wounds causes chronic ulceration. This phenomenon frequently occurs in areas not influenced by repetitive stretch stimuli, such as sacral decubitus pressure ulcers. There appears to be a strong clinical correlation between wound contracture and repetitive mechanical stretching. In the present study, we discovered that repetitive mechanical stretching activates TRPC3 channels in fibroblasts, which leads to increased production of fibronectin, a key regulator of wound contraction. The following observations support this conclusion: (a) Human hypertrophic scars obtained from areas exposed to repetitive stretching demonstrated higher expression levels of the TPRC3 channel compared to normal skin; (b) human primary fibroblasts express more TRPC3 channels when they are stretched; (c) TRPC3 overexpressing fibroblasts show more contractile activity compared to control fibroblasts; (d) TRPC3 overexpressing fibroblasts produce more fibronectin in response to mechanical stretching stimuli; (e) When TRPC3 overexpressing fibroblasts were stretched, transcriptional factor NFκB, a regulator of fibronectin expression, was more active than in control cells; (f) The rate of wound closure in mice transplanted with TRPC3 overexpressing cells was enhanced compared to the rate of wound closure in control mice. Taken together, these results indicate that TRPC3 is a mechanical force transducer that increases fibronectin production via the NFκB pathway in response to repetitive stretching. These results suggest that TRPC3 may play a significant role in wound contraction.

The effect of mechanical forces on cells has been well documented^28^,^36^,^37^,^38^,^39^. It is well known that focal adhesion proteins can serve as mechanosensitive elements that enable mechanical communication between cells and the extracellular matrix^40^. The activation of the Rho family of GTPase proteins in response to mechanical stretching has also been well established^41^,^42^,^43^. Among the key players involved in mechano-transduction, the canonical TRPC channels have emerged as important molecules that activate several intracellular signal transduction pathways in response to mechanical stimuli^29^,^44^,^45^. It has been shown that the TRPC channels and their downstream calcineurin/NFAT pathways are responsible for the process of pathologic cardiac remodeling^29^,^31^,^33^,^34^,^46^. In idiopathic pulmonary arterial hypertension, enhanced expression of TRPC has been reported^47^. However, in terms of the link between repetitive stretching forces and scar contracture formation, the role of TRPC channels has not been previously reported. The immunohistochemical and qRT-PCR data presented in this study demonstrate that TRPC3 expression is dramatically increased in contracted hypertrophic scar tissue, suggesting an association between TRPC3 and pathologic scar formation.

It is well established that TRPC-mediated calcium influx directly activates calcium sensitive phosphatase calcineurin, which subsequently activates transcriptional factor NFAT. In this study, however, we further demonstrated that the NFκB pathway can also be activated via TRPC3 in response to mechanical stretching. The NFκB family of transcription factors consists of homo- or hetero dimeric subunits of the Rel family, including RelA (p65), p50, p52, RelB and c-Rel. NFκB belongs to the category of “rapid-acting” primary transcription factors, which quickly respond to various cellular stimuli, such as reactive oxygen species, tumor necrosis factor alpha or interleukin 1 beta. A number of studies have reported that the concentration of intracellular calcium also regulates NFκB activation. Tabary *et al*. showed calcium-dependent regulation of NFκB activation in the airway epithelial cells of cystic fibrosis patients^26^. Zhu *et al*. reported that the calcium oscillation frequency regulates trascriptional activity of NFκB in the human bronchial epithelial cell line, 16-HBE^27^. Palkowitsch *et al*. demonstrated the calcium-calcineurin dependence of T cell receptor-induced NFκB activity^48^.

TRPC family members can be transcriptionally induced and/or directly activated by G-protein coupled receptor signaling through diacylglycerol. Furthermore, TRPC channels are susceptible to opening in the setting of low intracellular calcium stores or by stretching of the plasma membrane^30^,^49^. Although TRPC channels form homo- or heterotetramers, it is reported that overexpression of any one subunit alone can enhance Ca2+ currents^34^. In this study, we generated TRPC3 overexpressing fibroblasts and demonstrated that the degree of calcium influx in TRPC3 overexpressing fibroblasts in response to repetitive mechanical stretching is stronger than that of control fibroblasts. The stepwise increase of a fluorescent calcium indicator strongly suggested the accumulation of cytoplasmic calcium upon repetitive stretching, which is consistent with the results of NFκB activation in TRPC3 overexpressing cells. This mechanism may cause continuous activation of NFκB in cutaneous wounds subjected to repetitive stretching.

Fibronectin, which is known to be involved in the wound healing process and scar contracture, is regulated by NFκB. Fibronectin deposition is apparent in fibrotic lesions in many organs^35^,^50^,^51^,^52^. Fibronectins are high molecular weight glycoproteins that accumulate in tissue as an ECM component. It is also well known that fibronectin is not simply a scaffolding material of the ECM, but also serves as a signaling molecule that regulates cell adhesion, growth, migration and differentiation^8^. The cellular responses elicited by fibronectin are mediated via surface integrins or other receptors such as TLR4^11^,^53^. During the wound healing process, up-regulation of fibronectin in fibroblasts is seen^8^,^54^,^55^. Unregulated fibronectin expression can lead to uncontrolled wound contraction. Indeed, in hypertrophic scar tissue, fibronectin accumulates and is concentrated in nodular areas^35^,^54^,^55^. Moreover, Fibronectin gene transcription is enhanced in keloid fibroblasts^56^. In the present study, we show that TRPC3 is also distributed along the nodular areas of scar tissue, which is consistent with the distribution of fibronectin. This finding also supports that TRPC3 plays a role in the formation of pathological scar contracture.

The regulation of fibronectin gene transcription is under complex control. For example, cAMP-responsive elements mediate Fibronectin gene expression in response to cAMP^57^. The Sp1 transcription factor regulates the transcription of Fibronectin by binding to Sp1 site^20^. EGR-1 has been shown to induce the expression of Fibronectin in fibrosarcoma and glioblastoma cells^17^,^58^. NFκB is a fibronectin transcriptional regulator that binds to the kappa-B motif (GGGACTTTCC). Lee *et al*. reported that NFκB activates fibronectin gene expression in rat hepatocytes^16^. In fibroblasts from abnormal wounds (keloid tissue), NFκB was shown to be activated^25^. The result of our gel shift assay demonstrated that NFκB was activated in TRPC3 overexpressing fibroblasts when they were stretched. This data indicates that fibronectin expression was upregulated in TRPC3 over-expressing fibroblasts in response to mechanical stretching and that the process was mediated by NFκB. Interestingly, You *et al*. showed that fibronectin activates NFκB in dermal fibroblasts^59^, which implies the formation of loop where NFκB and fibronectin activate each other.

**Figure 8:**
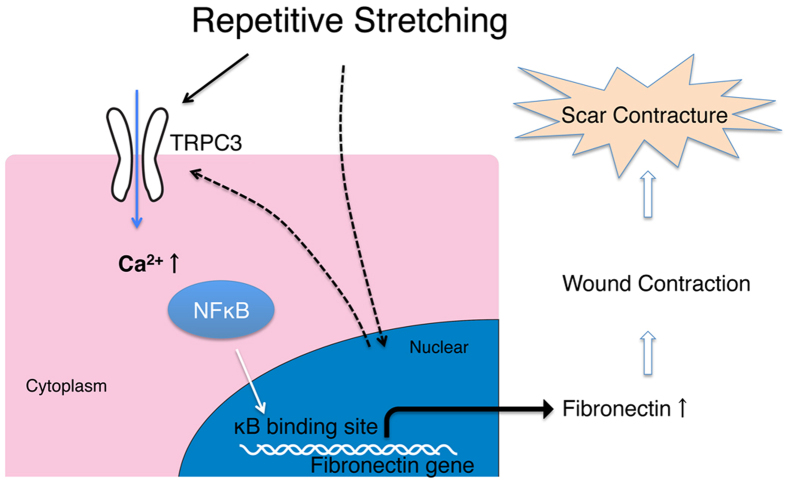
Proposed model for the role of TRPC3 in hypertrophic scar contracture. TRPC3 gene transcription is upregulated by mechanical stretching forces. Increased TRPC3 expression leads to increased calcium influx, which induces NFκB phosphorylation. Activated NFκB translocates into the nucleus and promotes Fibronectin gene expression, which ultimately enhances wound contraction.

In conclusion, the TRPC3 channel is a potential mechanical force transducer that plays an important role in the pathogenesis of hypertrophic scarring. In the setting of repetitive mechanical stretching of cutaneous wounds, TRPC3 expression is increased, which results in increased calcium influx and the activation of NFkB. This in turn causes enhanced production of fibronecin, which may account for hypertrophic scar contracture (Fig. 8). Although the mechanism by which the transcription of TRPC3 in stretched wounds is upregulated remains to be determined, these findings might offer a new target for developing therapeutic interventions to prevent and treat fibrotic diseases and hypertrophic scarring.

## Methods

### Cell culture

PlatE packing cells were provided from Dr. Kitamura at the University of Tokyo. NIH3T3 fibroblasts were a gift from Dr. Fukuhara at the Graduate School of Medicine at Osaka University. Cells were cultured in Dulbecco’s Modified Eagle’s Medium (DMEM; Sigma, MO, USA) supplemented with 10% fetal bovine serum (FBS) and 1% antibiotic solution (penicillin (100 U/ml and) -streptomycin (100 ug/ml) Mixed Solution, Nacalai Tesque, Kyoto, Japan) in an incubator set at 37 °C, 5% CO_2_. Normal human skin fibroblasts were obtained from normal adult human skin samples that were discarded after scar revision surgery (performed at Hyogo College of Medicine). The normal skin samples were obtained from non-scar regions of the discarded skin samples. The isolated fibroblasts were cultured in DMEM containing 10% FBS and antibiotics until they become confluent.

### Generation of TRPC3 overexpressing fibroblasts

PMXs-IRES-GFP plasmids were provided from Dr. Kitamura at the University of Tokyo. The mouse cDNA clone TRPC3 was obtained from Origene Technologies Inc. (MD, USA). We constructed a retroviral plasmid using the pMXS-IRES-GFP construct by incorporating the TRPC3 gene into the plasmid. Then, the plasmids were transfected into packaging cells; Plat E, which produced retrovirus containing both TRPC3 and GFP. Transfection was carried out using Lipofectamin LTX (Life Technologies, CA, USA). Mouse skin fibroblasts (NIH3T3) were infected with the virus, which allowed them to express both TRPC3 and GFP. As a control, we transfected the virus that contained the vector alone into NIH3T3 cells. Then, GFP positive cells were collected using a cell sorter (FACS Aria, Becton,Dickinson, NJ, USA). We confirmed robust expression of TRPC3 in our product cells with both qRT-PCR and Western blot analysis, where an anti-TRPC3 antibody was applied to the cell lysate and a protein band corresponding to the size of TRPC3 was identified.

### Cyclic mechanical stretching

In each experiment, either primary human dermal fibroblasts, TRPC3 overexpressing fibroblasts, or empty vector transfected control fibroblasts were seeded on a Type I collagen-coated silicon chamber (3 × 3 cm) at a density of 8 × 10^5^ cells/chamber. After allowing the cells to attach for 24 h, the culture medium was replaced with DMEM supplemented with 0.5% BSA and 1% antibiotic solution. Cells were incubated in the serum-starved condition for 24 h. Then, the fibroblasts were mechanically stretched to 20% strain for the indicated times at a frequency of 10 cycles/min. Each stretching cycle consisted of 2 seconds of strain and 2 seconds of relaxation. The stretching was conducted using a cell stretcher (STB-140-10, STREX, Osaka, Japan) that was placed in an incubator set at 37 °C and 5%CO_2_.

### Calcium imaging

Either TRPC3 overexpressing fibroblasts or empty vector transfected control fibroblasts were seeded and cultured in silicon chambers, as described above. After 24 hours of incubation, the culture media was replaced with Hank’s solution containing 5 μM Fluo4-AM (Dojindo Molecular Technologies, Inc, Kumamoto, Japan) and 0.02% Pluronic F-127 (ANA Spec, Fremont, CA, USA). The cells were incubated at 37 °C and 5%CO_2_ for 20 minutes. Then, the silicon chambers were set on the cell stretcher, which was placed under fluorescent microscopy (Nikon Ti, Nikon, Tokyo, Japan). Real-time calcium influx was observed while stretching (20% stretch at a frequency of 20 cycles per minutes). Live images were captured for several minutes using the NIS Elements (Nikon, Tokyo, Japan).

### Immunocytochemistry and Immunohistochemistry

Three different human hypertrophic scar samples and corresponding adjacent normal skin samples were obtained during scar revision surgeries performed at the Hyogo College of Medicine. The samples were soaked in O.C.T Compound (SAKURA, Tokyo, Japan) at room temperature and immediately placed into liquid nitrogen. Sections were harvested at a thickness of 12 μm. For immunohistochemistry, tissue sections were blocked using normal goat serum and incubated with rabbit anti-TRPC3 antibody (Alamone Labs, Jerusalem, Israel) at 1:100 dilution for 1.5 hours at room-temperature. Sections were incubated with a biotinylated goat anti-rabbit secondary antibody for 30 minutes and developed with ABC complex (Vectastain ABC System, Vector Labs, CA, USA). Primary antibody binding sites were visualized using Peroxidase Stain DAB kit (Nakalai tesque, Kyoto, Japan). Sections were then counter-stained with hematoxylin. For immunocytochemistry, primary human fibroblasts were stretched for 24 hours as previously described. Then, either stretched or un-stretched cells were fixed with ice-cold 4% paraformaldehyde for 15 minutes. The bottom of the silicon chamber was then cut into small pieces (5 mm × 5 mm) and a piece of the chamber was attached to a slide glass. After blocking with normal goat serum, the cells were incubated with anti-TRPC3 antibody at 1:100 dilution for 1 hour at room temperature. Cells were then developed with Vectastain ABC system and DAB staining. Hematoxylin was used for counter-staining. For phospho-NFkB translocation immunocytochemistry, either control or TRPC3 overexpressing fibroblasts were stretched for 15 minutes and treated as mentioned above. After 30 minutes of blocking with 2% horse serum, the cells were incubated with either anti-phospho-NFkB antibody (Cell Signaling Technology, MA, USA) at 1:200 dilution or anti-NFkB antibody (Cell Signaling Technology, MA, USA) at 1:500 dilution for 30 minutes. Alexa Fluor 594 donkey anti-Rabbit IgG (Abcam, Cambridge, UK) at 1:250 dilution was used for the secondary antibody. Following staining, the cells were mounted with VECTASHIELD with DAPI (VECTOR Laboratories, CA, USA). For immunofluorescence with mouse wound tissue, mice were sacrificed at 9 days post-wounding. Wound tissue was harvested and soaked in 4% paraformaldehyde at 4 °C overnight and embedded in paraffin. The tissue samples were cut to a thickness of 4 mm. The specimens were deparaffinized and retrieved with Target Retrieval Solution (DAKO, Glostrup, Denmark), followed by blocking and permeabilization with 2% horse serum and 0.05% Triton X100 in PBS. The specimens were then incubated with antibodies to GFP (1:250), TRPC3 or Fibronectin (1:100), followed by AlexaFluor labeled antibodies (Abcam, Cambridge, UK). Nuclei were identified with DAPI. Immunofluorescence was evaluated under an immunofluorescence microscope (AX80, Olympus, Tokyo, Japan).

### Quantitative real-time PCR

Total RNA from human skin tissue and cultured cells was isolated from human skin tissue samples with Isogen (Nippon Gene, Tokyo, Japan) according to the instructions provided by the manufacturer. The mRNA concentration was measured using a Nanodrop spectrometer (NanoDrop Technologies, USA). Aliquots of 900 ng of mRNA were reverse-transcribed to complementary DNA using PrimeScript RT Master Mix (TAKARA, Shiga, Japan). Quantitative real-time PCR was performed using SYBR Green PCR Master Mix (Applied Biosystems, CA,USA) and the 7900 Real-Time PCR System (Applied Biosystems, CA, USA). The following primers were used: Human TRPC3(F); 5′-AGC CGC AGC AGT ATG TGG ATA-3′, Human TRPC3(R); 5′-GGG AGC CAT TTG TCT CTA GCA TAG G-3′, Human cyclophilin(F); 5′-CAG ACG CCA CTG TCG CTT T-3′, Human cyclophilin(R); 5′-TGT CTT TGG AAC TTT GTC TGC AA-3′, Mouse Fibronectin (F); 5′-ACG TCA TTG CCC TGA AGA AC-3′, Mouse Fibronectin (R); 5′-CCA TTT TCG GTG TCA TAC CC-3′, Mouse 18S (F); 5′-GTG GAG CGA TTT CTG TGG TT-3′, Mouse 18S (R); 5′-CGC TGA GCC AGT CAG TGT AG-3′. Cyclophilin and 18S were used for the internal standards.

### Western blot analysis

Cells were stretched as described previously for the indicated time. The TRPC3 specific inhibitor Pyr3 (Sigma Aldrich, MO, USA), the NFkB inhibitor Wedelolactone (Sigma Aldrich, MO, USA), or the same amount of vehicle (DMSO) was added to the media 5 minutes prior to stretching. Then, the cells were washed with ice-cold PBS and the whole cell lysate was harvested with RIPA buffer (Nacalai Tesque,Kyoto, Japan) according to the manufacturer’s instructions. The protein concentration was measured and standardized using the BCA protein assay kit (Thermo scientific, MA, USA). After treatment with 2x Laemmli sample buffer (BIO RAD, CA, USA), 2 μg of protein (for IκB blotting, 20 μg of protein) was loaded on a 10% sodium dodecyl sulfate-polyacrylamide gel and then transferred to a PVDF membrane (BIO RAD). The membranes were blocked with 2% ECL prime blocking agent (GE Healthcare, Buckinghamshire, UK) diluted in 0.05% TTBS. Membranes were then incubated with primary anti-fibronectin antibody (Sigma Aldrich, MO, USA) diluted at 1:500, or anti- NFκB p65 antibody (Cell Signaling, MA, USA) diluted at 1:500, or anti-Phospho- NFκB p65 (Ser536) antibody (Cell Signaling, MA, USA) diluted at 1:200, or anti- IκB antibody (Cell Signaling, MA, USA) diluted at 1:1000, or anti-Phospho- IκB antibody (Cell Signaling, MA, USA) diluted at 1:500. Then, the membranes were incubated with anti-rabbit IgG, horseradish peroxidase-conjugated secondary antibody, followed by detection of immunoreactivity with enhanced chemiluminescence (ECL Prime Western Blotting Detection Reagent, GE Healthcare, Little Chalfont, UK)). Anti-MAPK antibody was used as an internal control^60^.

### Fibroblast Populated collagen Lattice

Either TRPC3 overexpressing fibroblasts or vector-transfected control fibroblasts were embedded in type I collagen at a final concentration of 5 × 10^5^ cells/500 μl and a collagen concentration of 1.5 mg/ml. A volume of 500 μl per gel was placed into a 24-well plastic culture plate, which ensured that the gel would remain attached throughout the culture period. The cells were cultured in 10% FBS for 24 hours after polymerization, followed by serum starvation with 0.5% BSA DMEM for 24 hours. Then, the TRPC3 agonist OAG, the TRPC3 specific blocker Pyr3 or vehicle (DMSO) was added at the indicated concentration and the FPCLs were mechanically released from the plate. After 72 hours of incubation, the contraction of the gels was recorded with CanoScan (Canon, Tokyo, Japan) and analyzed with Image J software.

### Electromobility Shift Assay (EMSA)

For the isolation of nuclear proteins, NE-PER Nuclear and Cytoplasmic Extraction Reagents (Thermo Scientific, MA, USA) was used. The sequences for the 5′end biotin-labeled oligonulceotide probes were as follows; forward 5′-AGT TGA GGG GAC TTT CCC AGG C-3′, reverse 5′-GCC TGG GAA AGT CCC CTC AAC T-3′. After the protein concentration was measured with the BCA protein assay kit and standardized, EMSA was performed with the LightShift Chemiluminescent EMSA kit (Thermo Scientific, MA, USA) according to the manufacturer’s instructions. 5 ug of nuclear extracts were incubated with the probe at room temperature for 30 minutes. The reaction products were subjected onto 5% polyacrylamide gel electrophoresis in 0.5x TBE buffer and transferred to a nylon membrane. The DNA binding activity of NFkB was detected with chemiluminescence (Chemiluminescent Nulceic Acid Detection Module, Thermo Scientific, MA, USA) and analyzed using ImageQuant LAS4010 (GE Healthcare, Little Chalfont, UK).

### Wound contraction model *in vivo*: Open mouse wound

8 week old female Balb/c *nu/nu* mice were used. For cell transplantation, either TRPC3 overexpressing fibroblasts or empty vector transfected control cells were cultured as described above and trypsinized. The cells were then suspended in saline. The mice were anesthetized with pentobarbital. The dorsum of each mouse was sterilized with iodine and 70% ETOH. The fibroblasts were injected into the entire dorsal area of the dermal and subdermal layers of the mice. The application of the cells were either 1) 500 μl of the TRPC3 overexpressing fibroblasts solution 2) 500 μl of vector transfected control fibroblasts (1 × 10^6^ cells /mouse) or 3) 500 μl of normal saline.

At 10 days post-transplantation, open wounds were created. Mice were anesthetized with pentobarbital and the dorsum of each mouse was sterilized with iodine and 70% ETOH. All limbs were extended evenly so that the skin on the back became relaxed and symmetric. In order to make wound sizes as consistent as possible, the excision line was first traced with a 6 mm punch biopsy. The skin, including the panniculus carnosus, was carefully excised just above the myofascial layer with scissors. The wounds were washed with sterile saline and dressed with an anti-adhesive sheet (SILKYPORE DRESSING, Alcare, Tokyo, Japan). Each experimental group consisted of 3 mice (6 wounds). Wound size was measured at the indicated time points. Wound dressings were removed carefully with saline, so as not to change the wound size or shape. All limbs were extended evenly so that the skin on the back became relaxed and symmetric. A standard silicon ring was used as a reference frame for each photograph. Wound photographs were taken with a digital camera (D40, Nikon, Tokyo, Japan). The wound and reference ring areas were measured with Image J software (public software, NIH). The ratio of the wound area to the reference ring area was calculated, and the rate of wound closure was determined.

### Statistics

Results are presented as means ± S.D. of n observations. Data involving only two groups was analyzed using a two-tailed student t-test assuming unequal variances. When more than two experimental groups were compared, the data was analyzed using the Tukey-Kramer test to compare data between individual experimental groups. A p-value of <0.05 was considered to be statistically significant for all tests.

### Study approval

Tissue samples from patients were obtained with written informed consent. For animal studies, all animals were housed under standard conditions in the Hyogo College of Medicine animal facility under institution-approved guidelines. All procedures were approved by the Hyogo College of Medicine ethics committee (Approval #; 211012, 212014 and RIN-HI167) and performed in accordance with the approved guidelines.

## Additional Information

**How to cite this article**: Ishise, H. *et al*. Hypertrophic scar contracture is mediated by the TRPC3 mechanical force transducer via NFkB activation. *Sci. Rep*. **5**, 11620; doi: 10.1038/srep11620 (2015).

## Supplementary Material

Supplementary Information

Supplementary Video S1

Supplementary Video S2

Supplementary Video S3

Supplementary Video S4
