# Supplementary material for: Hypertrophic scar contracture is mediated by the TRPC3 mechanical force transducer via NFkB activation
Source: Sci Rep. 2015 Jun 25;5:11620. doi: 10.1038/srep11620 (PMC4479825; doi:10.1038/srep11620)
Supplement: Supplementary Information [file srep11620-s1.pdf]

Supplemental Information

Title

Hypertrophic scar contracture is mediated by the TRPC3 mechanical force transducer via NFkB activation

The Authors

Hisako Ishise, Barrett Larson, Yutaka Hirata, Toshihiro Fujiwara, Soh Nishimoto, Tateki Kubo, Ken Matsuda, Shigeyuki Kanazawa, Yohei Sotsuka, Kazutoshi Fujita, Masao Kakibuchi, Kenichiro Kawai

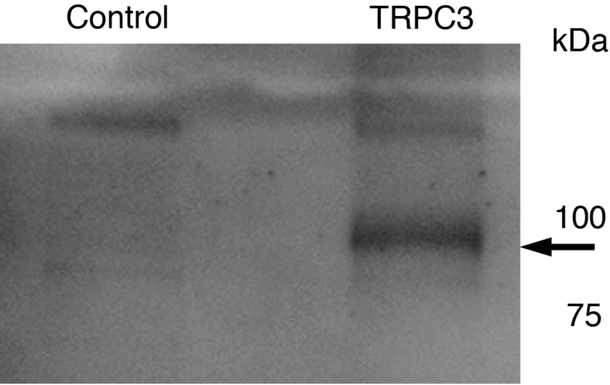

Figure S1

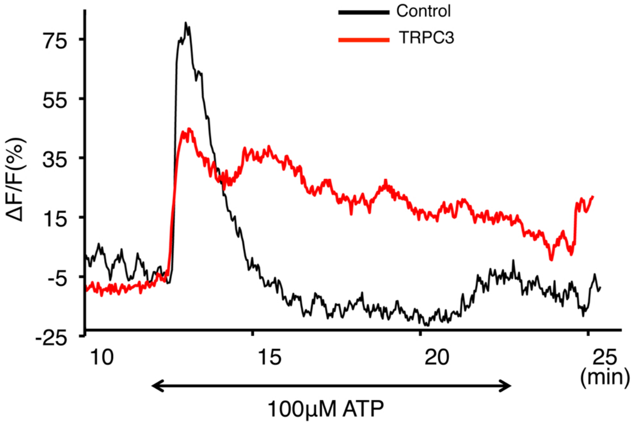

Figure S2

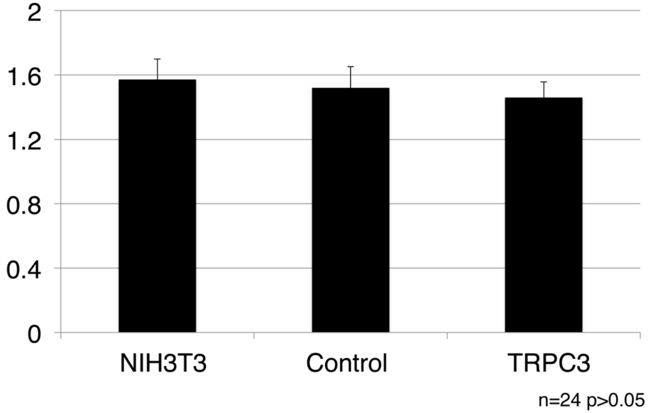

Figure S3

Anti FN1

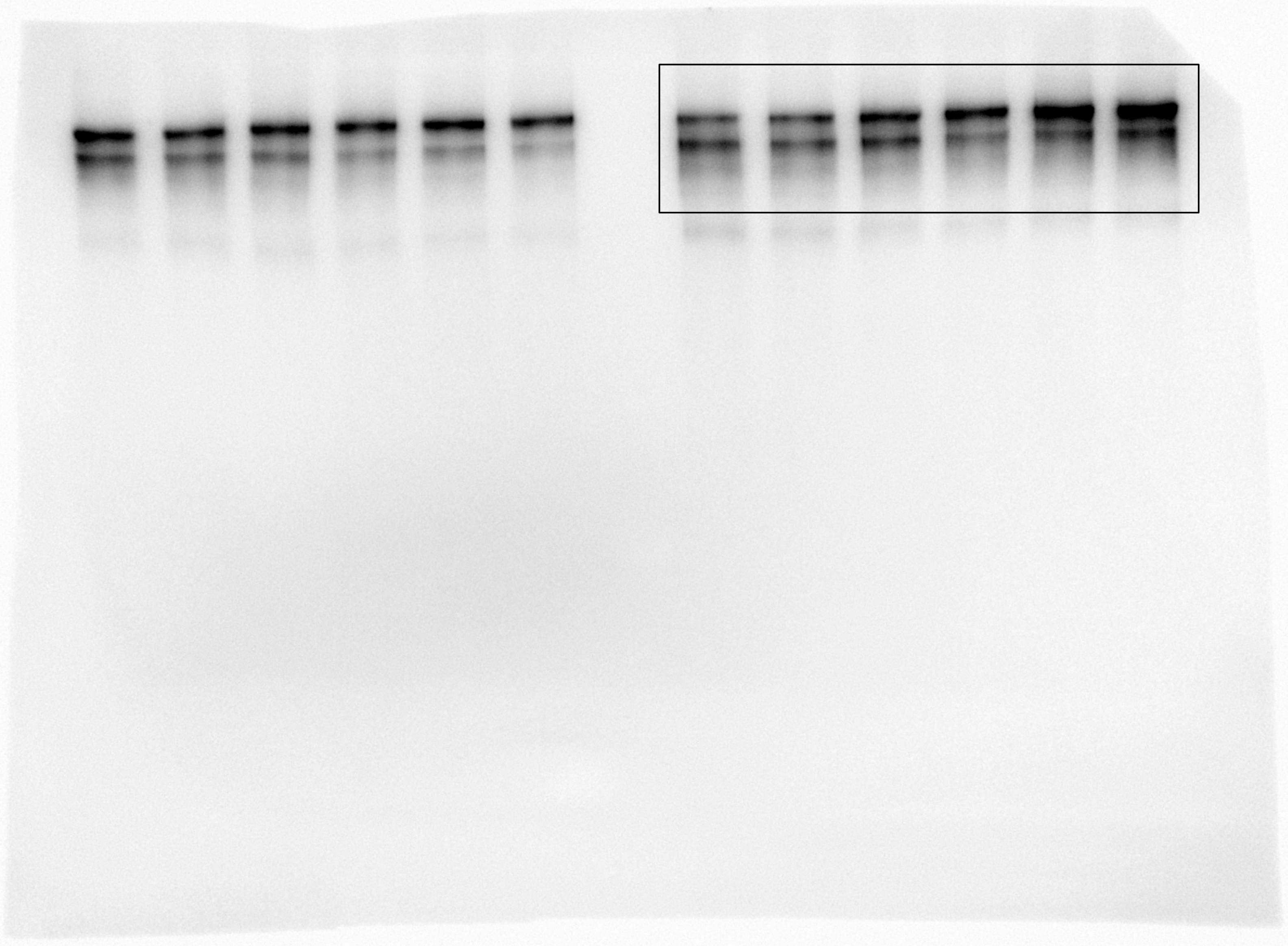

Figure S4

Anti MAPK

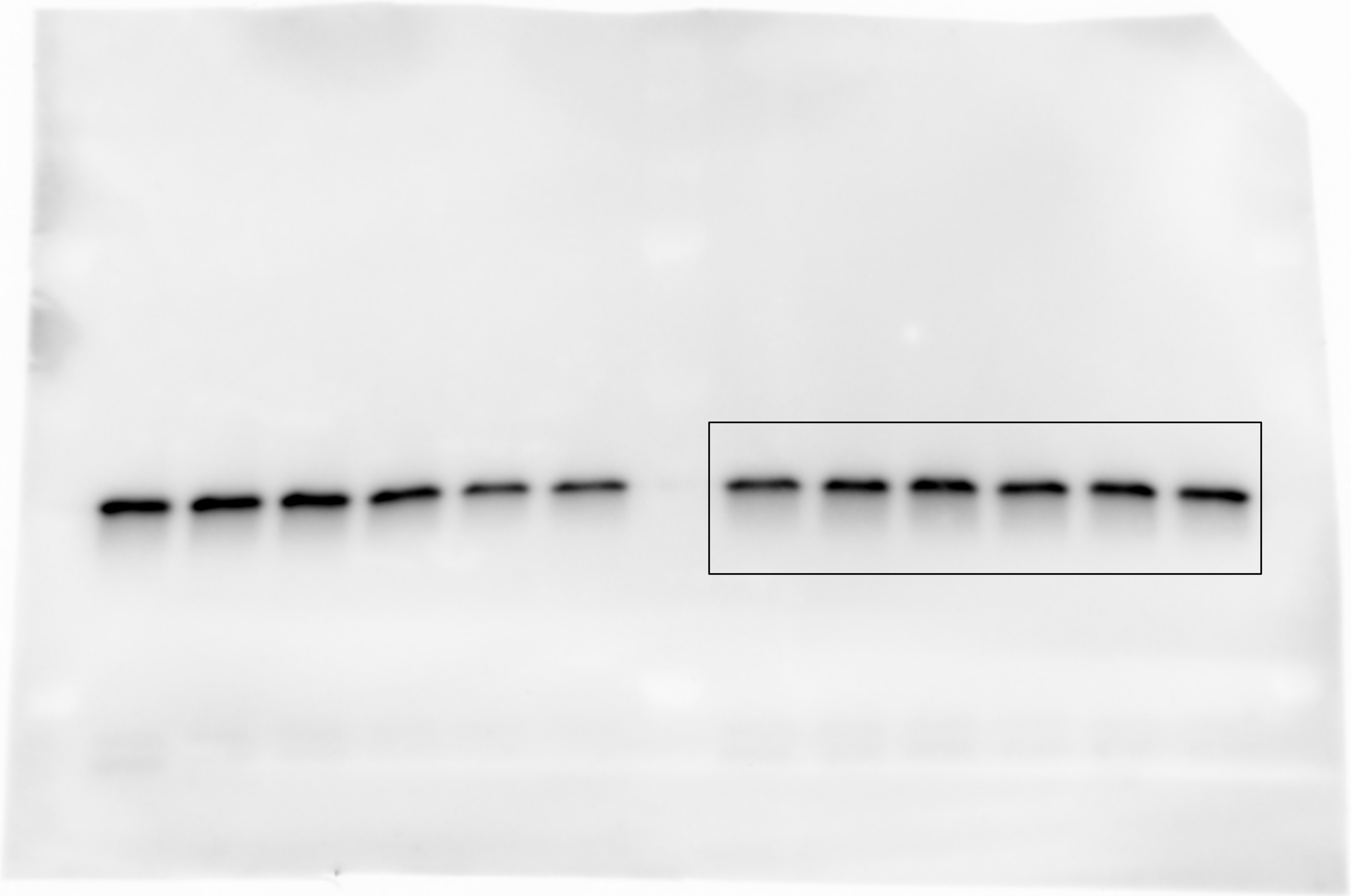

Figure S5

Anti FN1

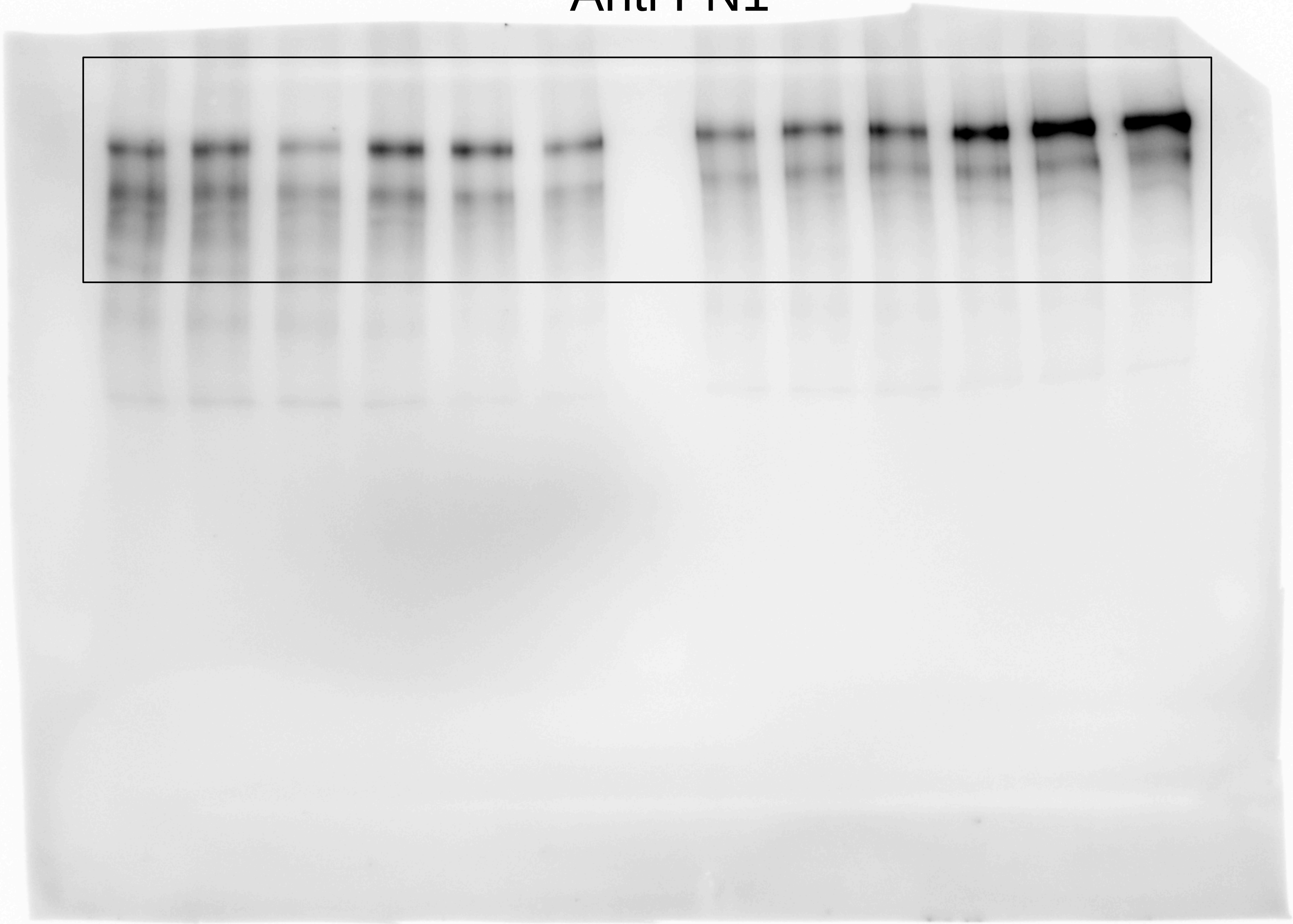

Figure S6

Anti MAPK

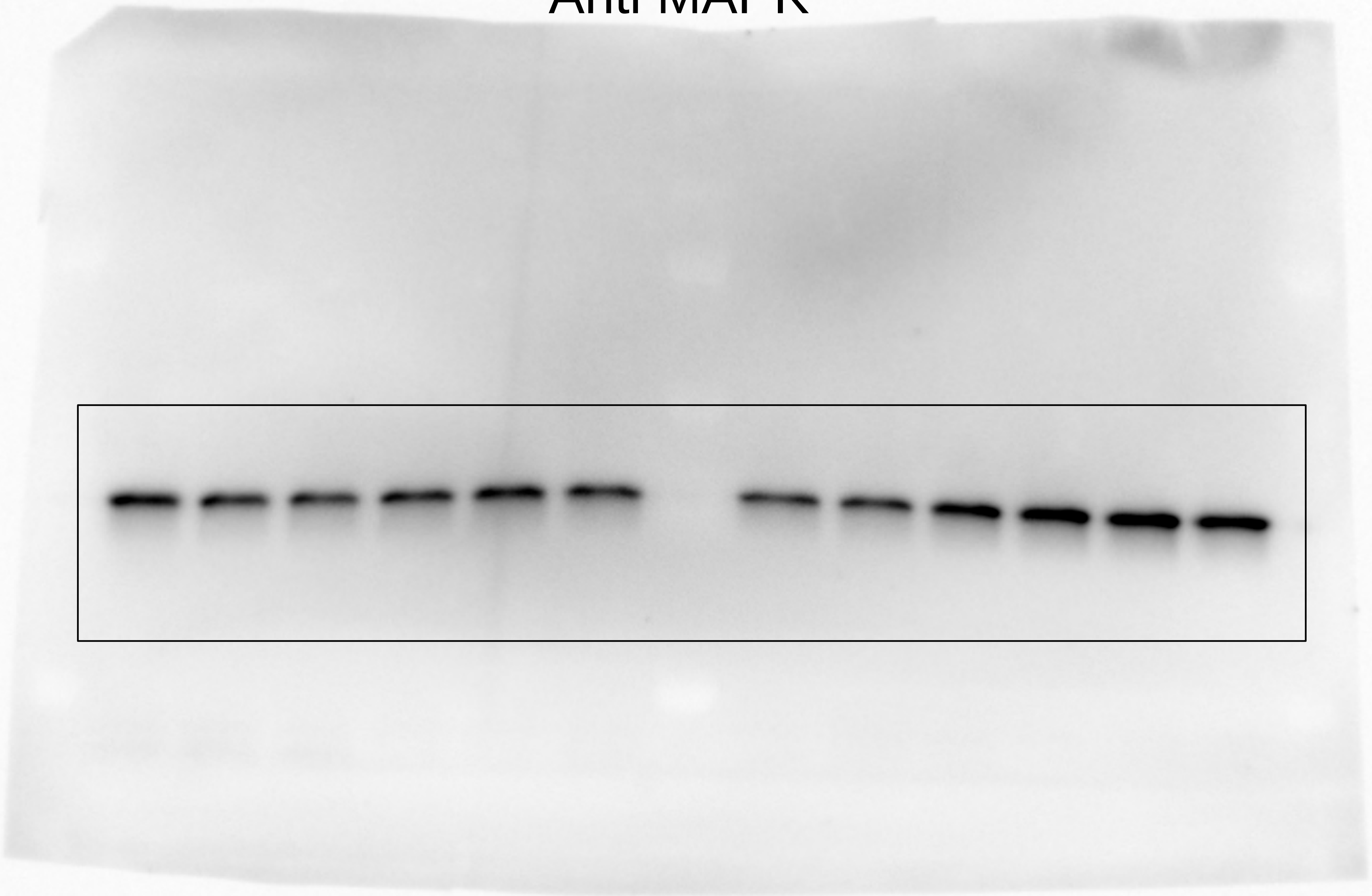

Figure S7

Anti FN1 Pyr3(-)

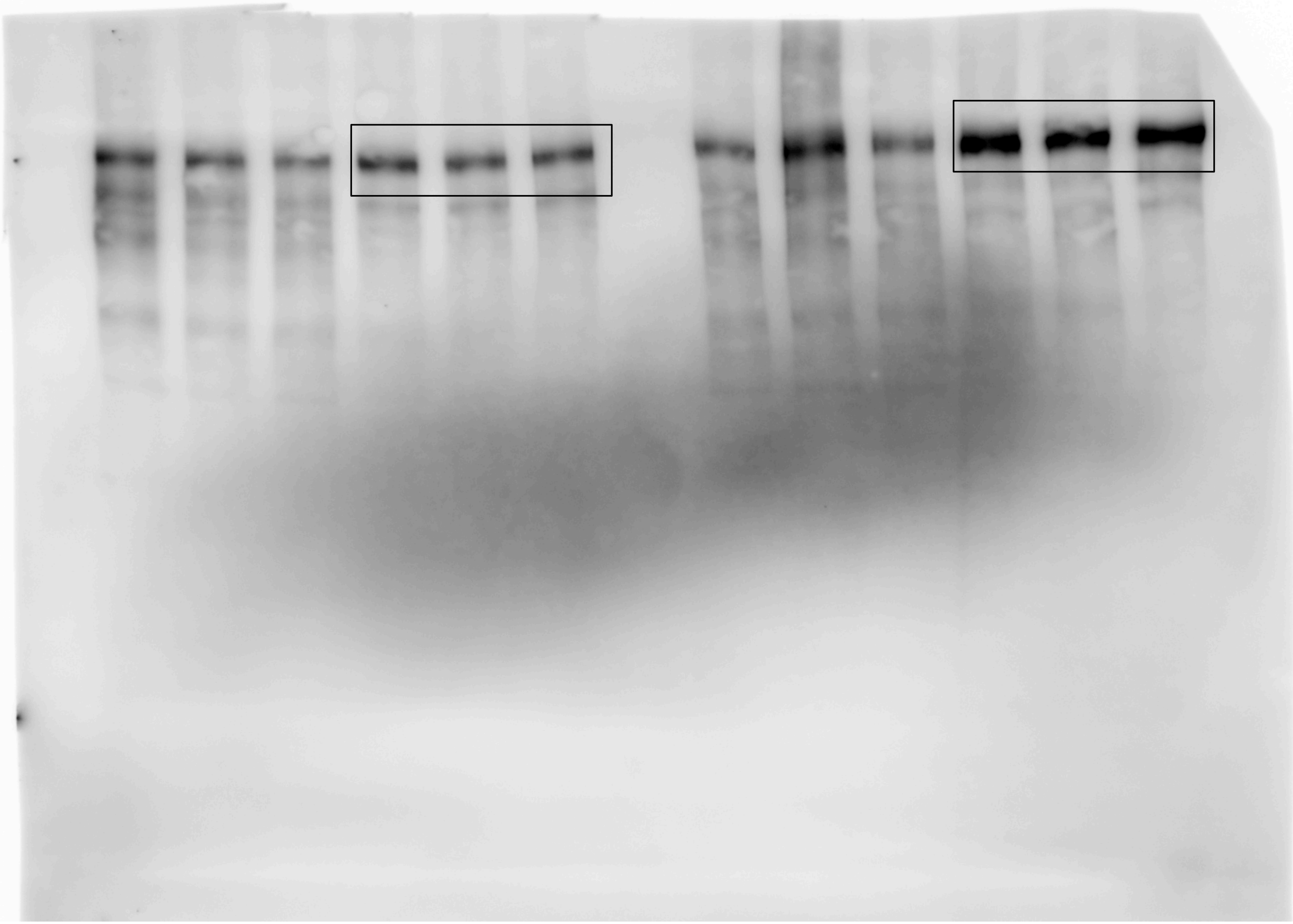

Figure S8

Anti FN1 Pyr3(+)

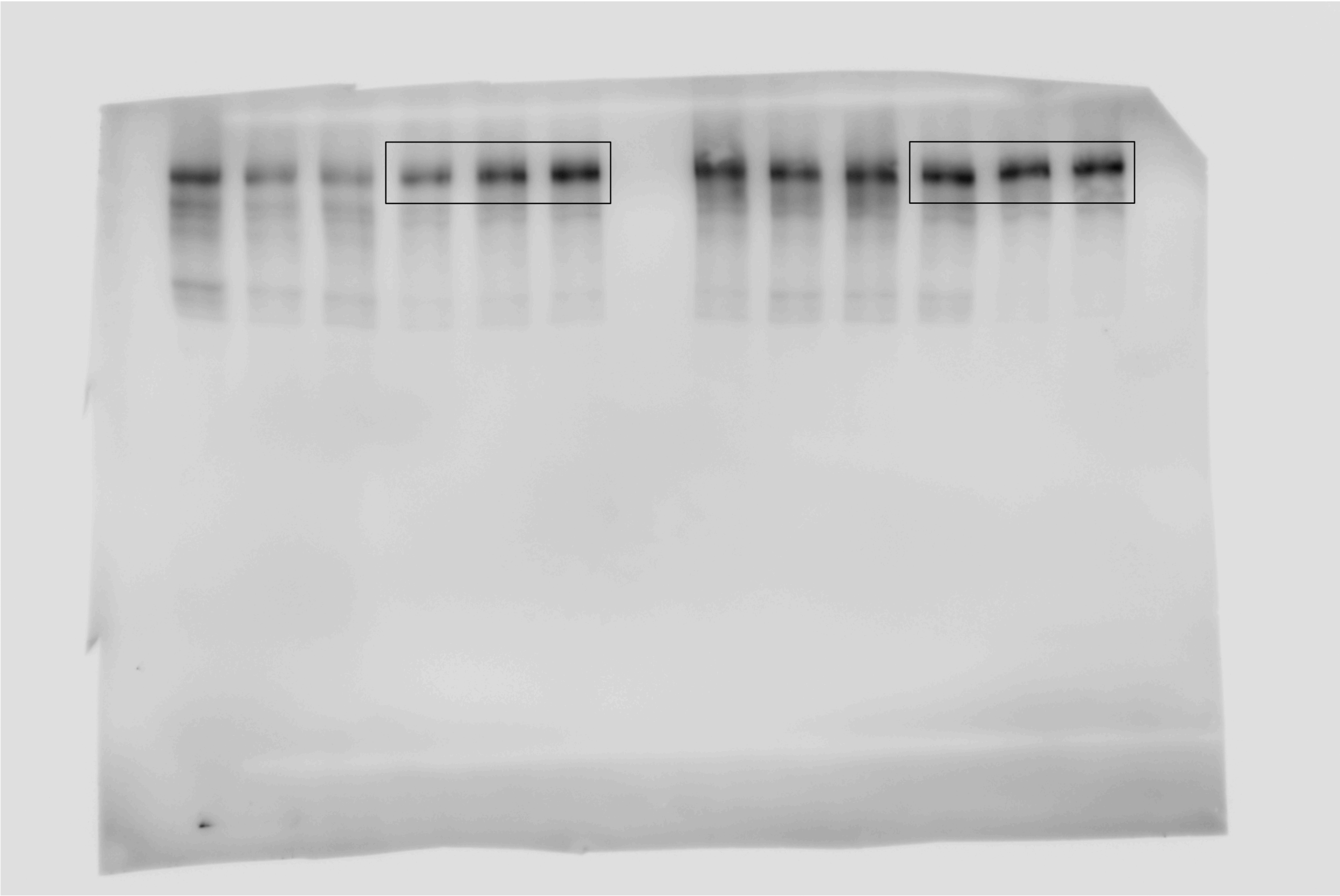

Figure S9

Anti MAPK

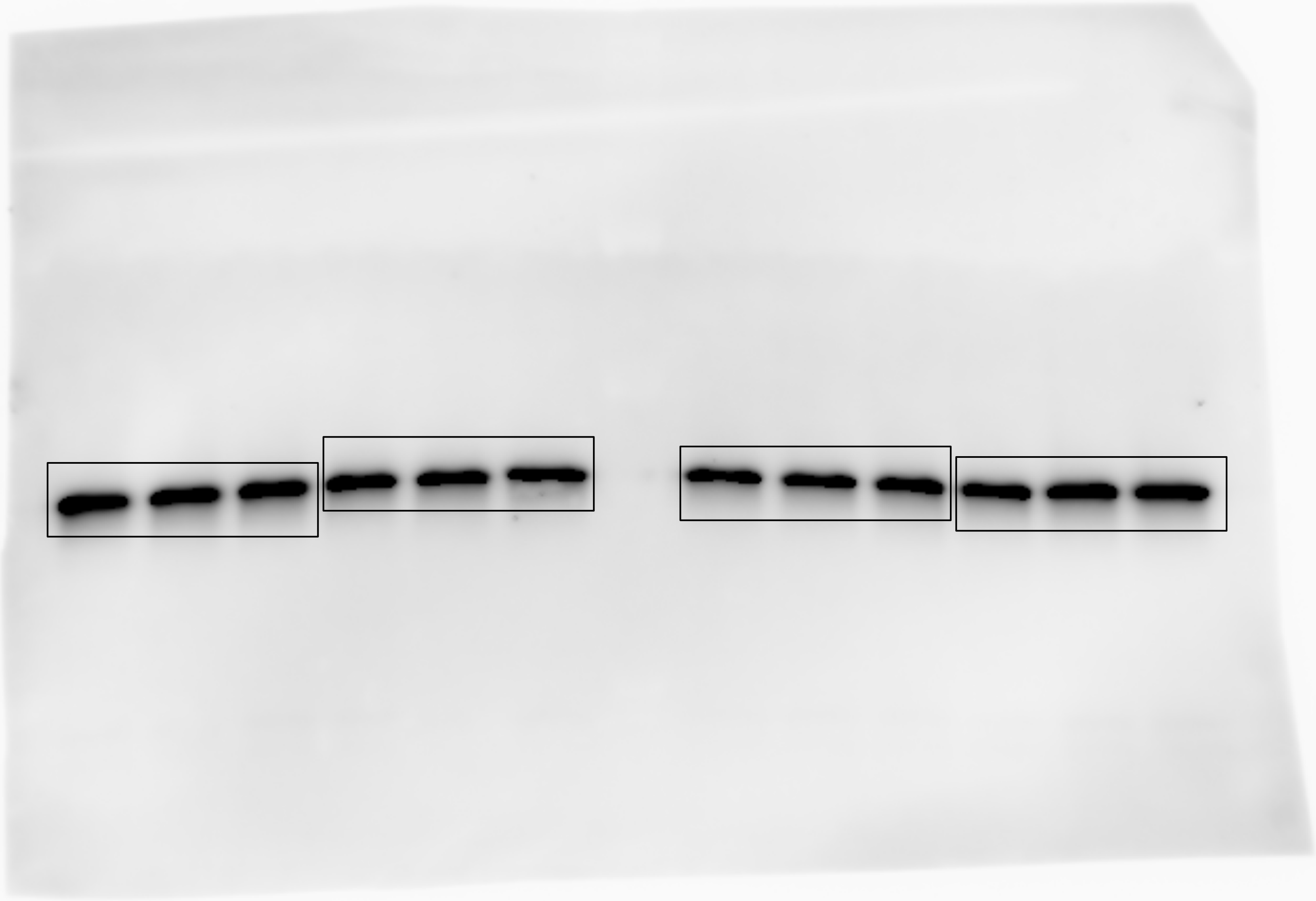

Figure S10

Anti FN1

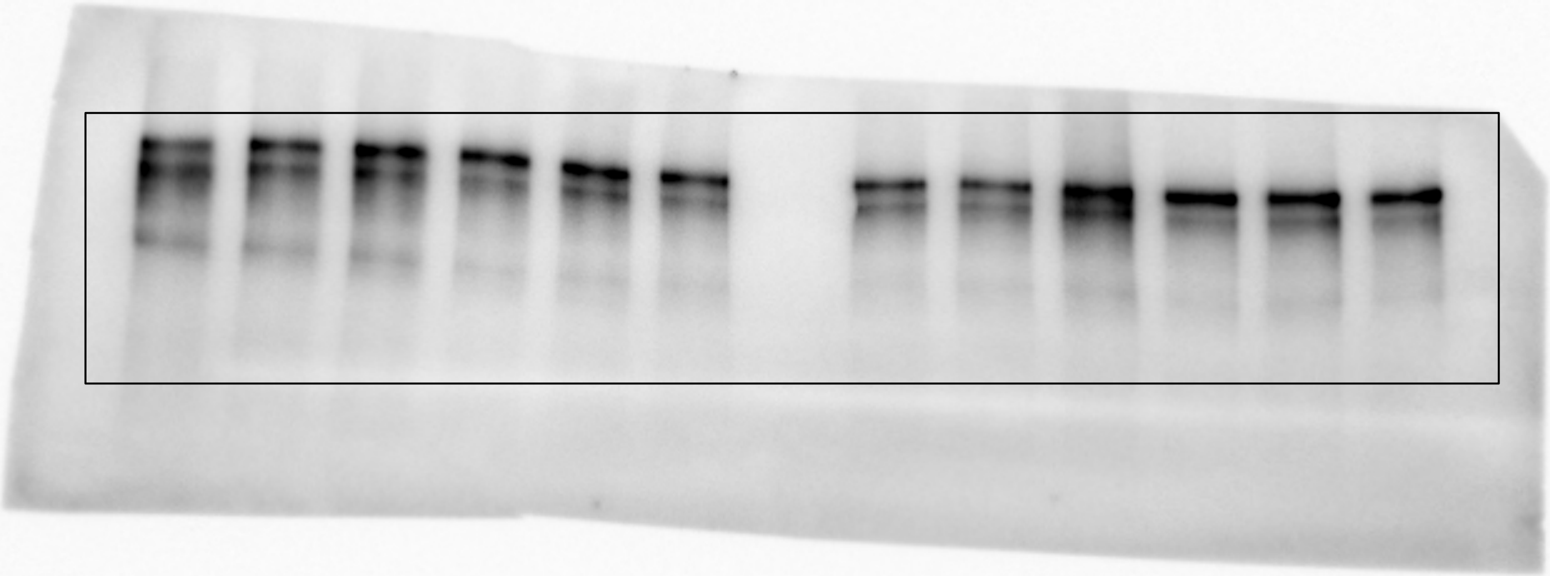

Figure S11

Anti MAPK

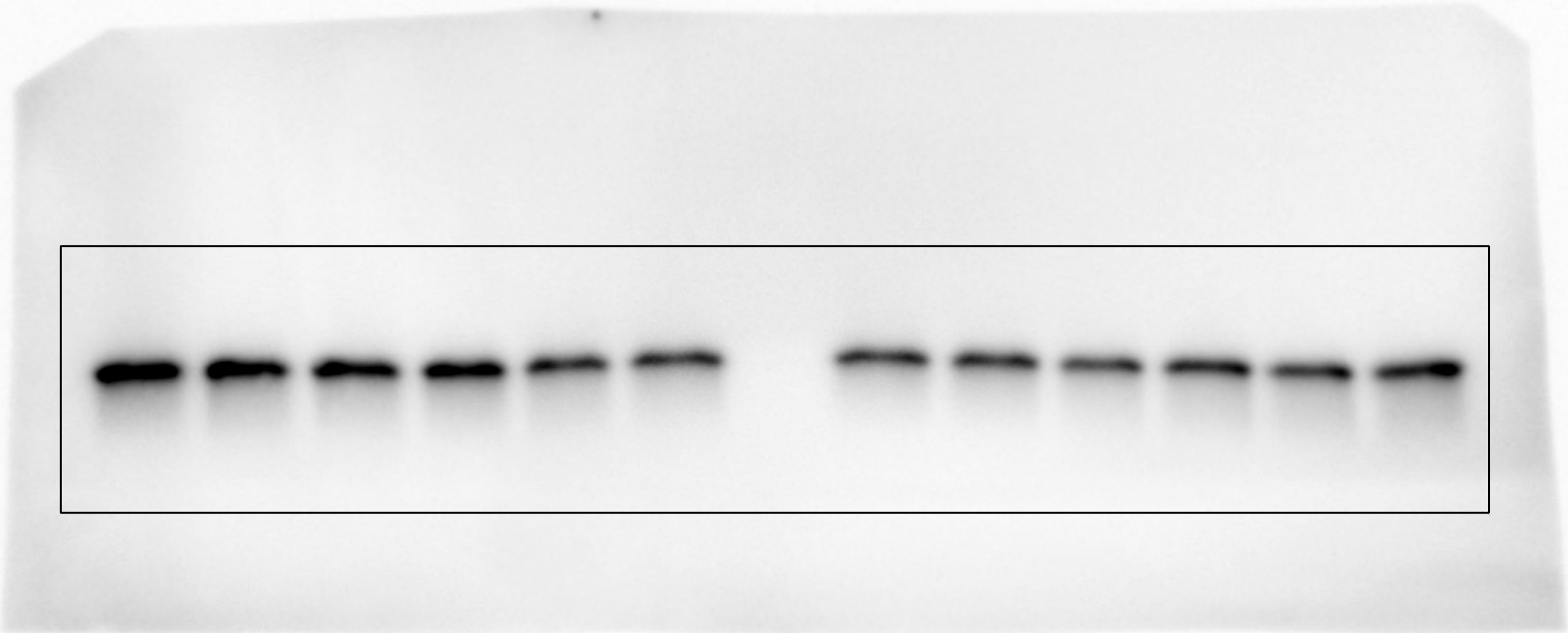

Figure S12

Anti p-NFκB

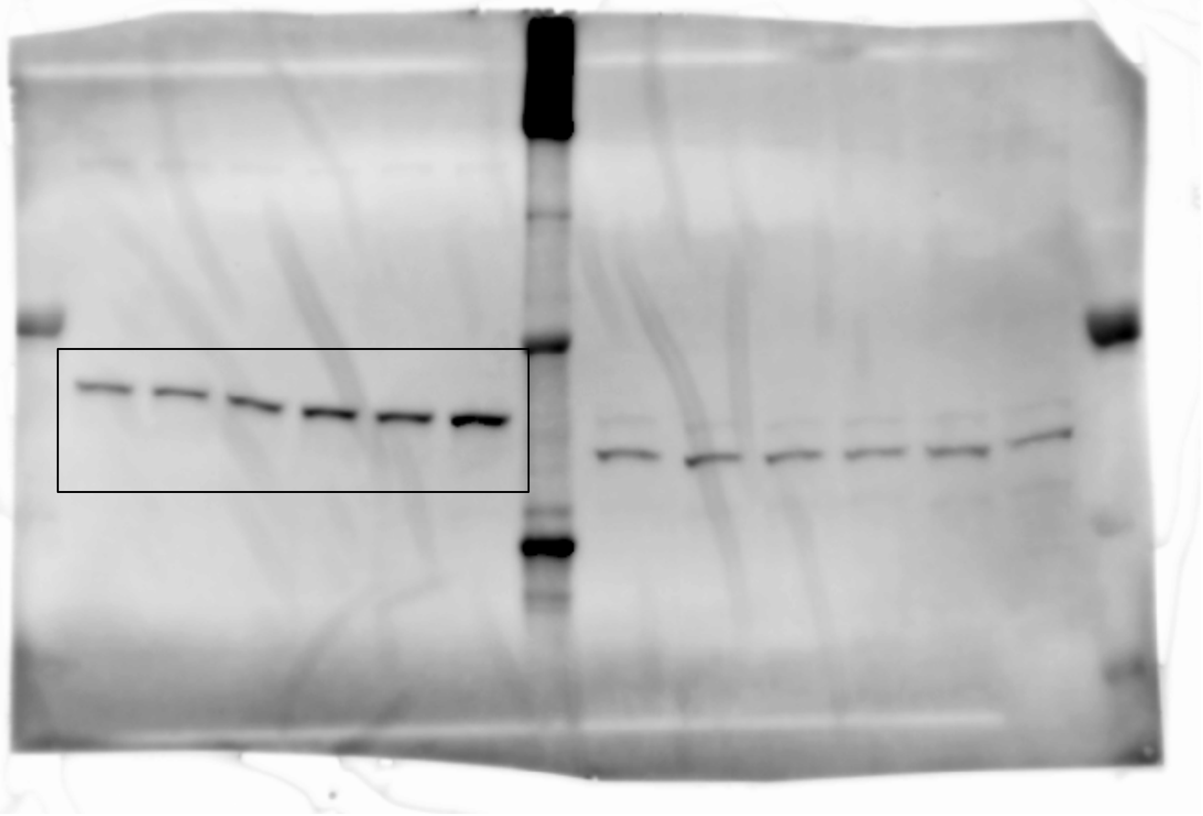

Figure S13

Anti NFκB

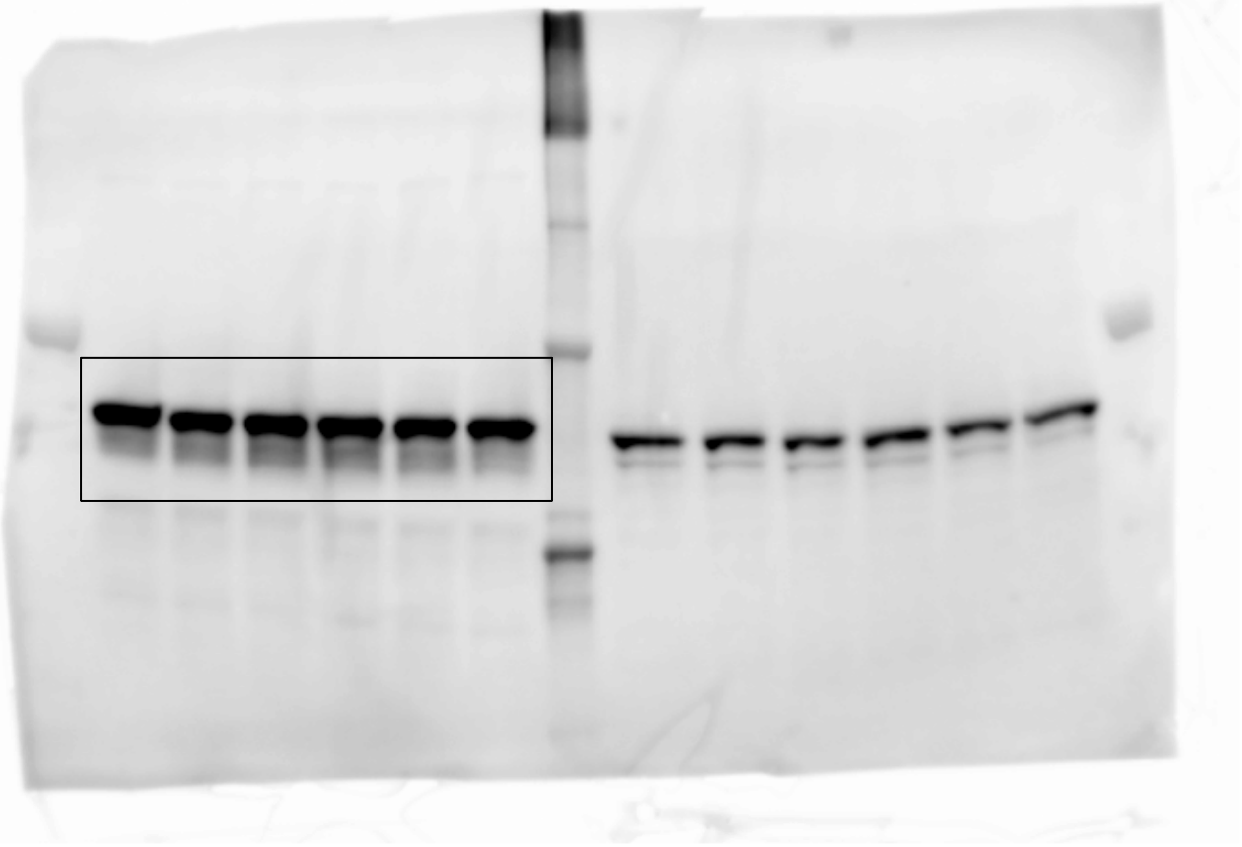

Figure S14

Anti p-NFκB

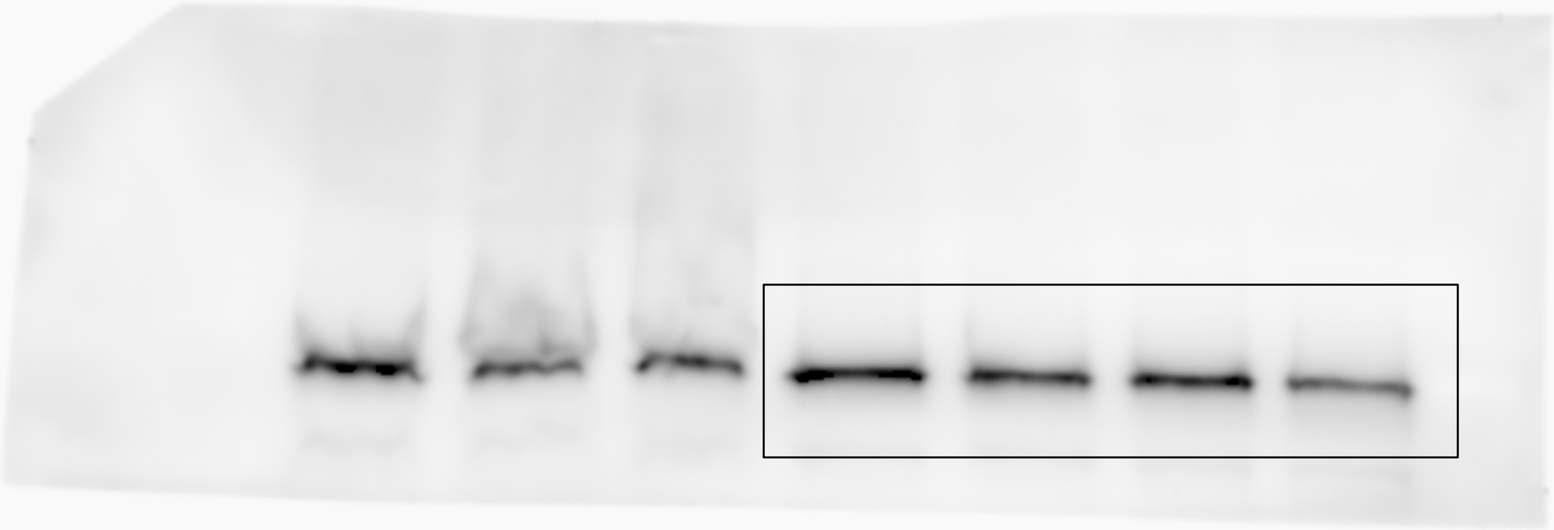

Figure S15

Anti NFκB

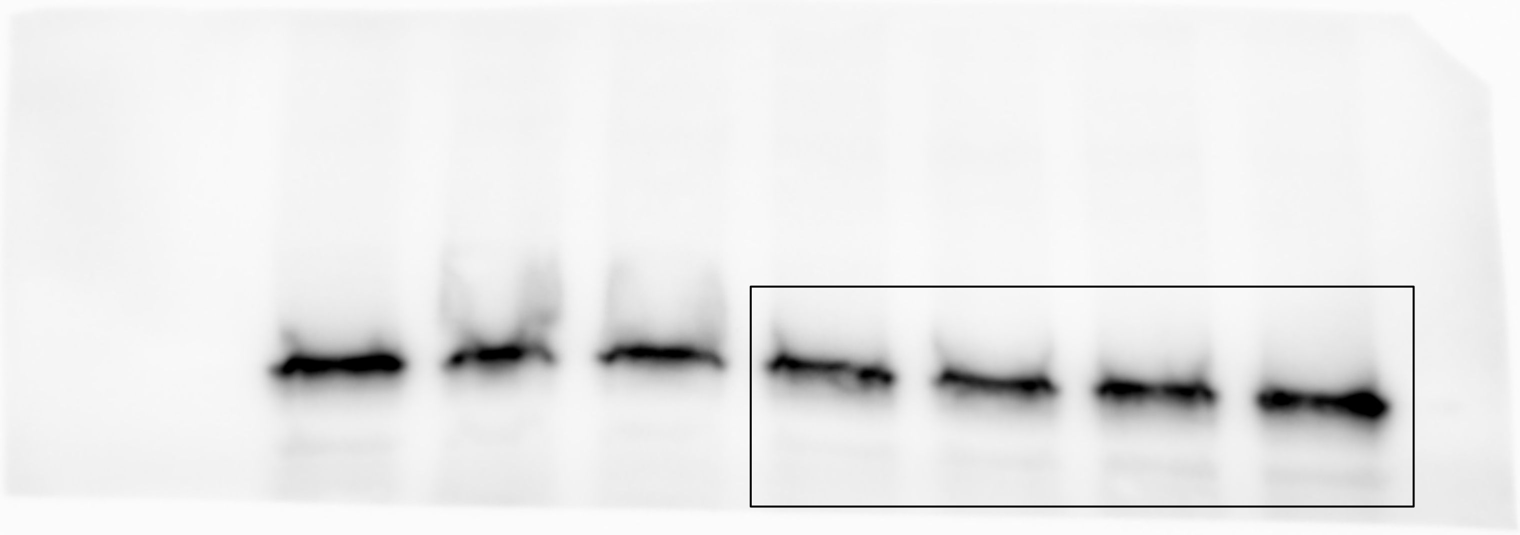

Figure S16

Anti p-IκB

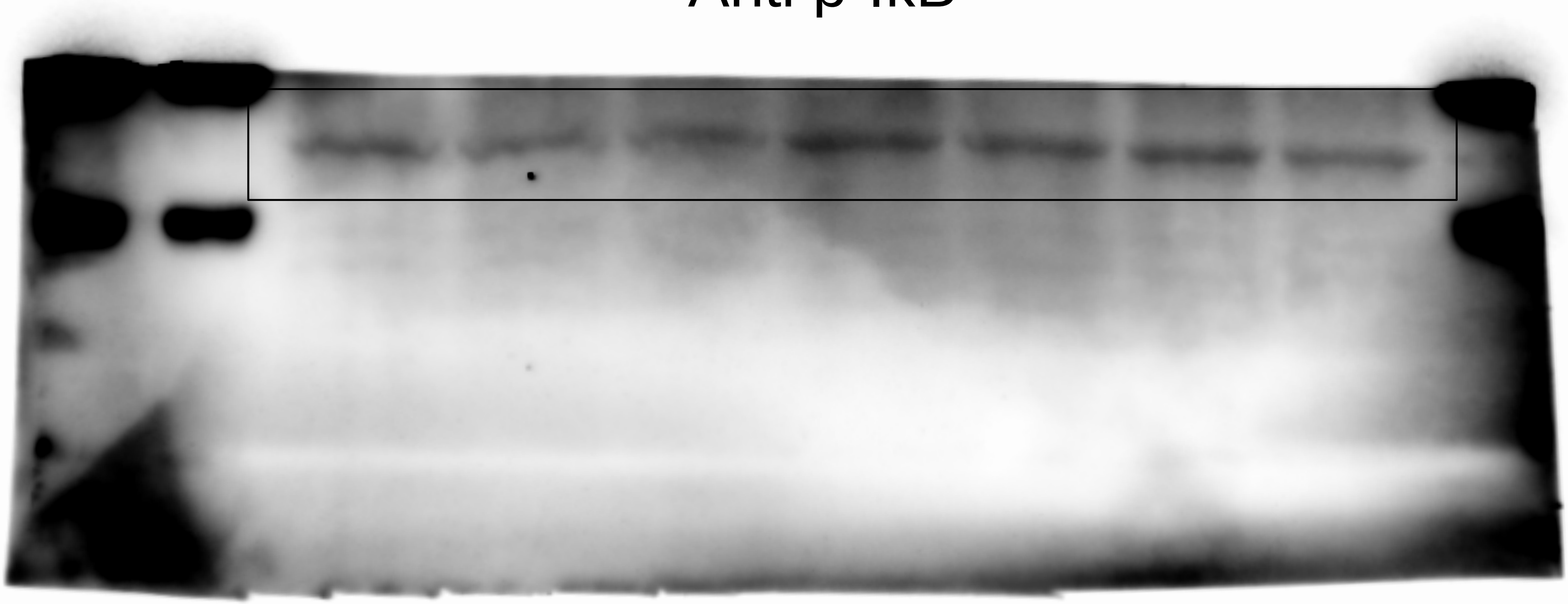

Figure S17

Anti IκB

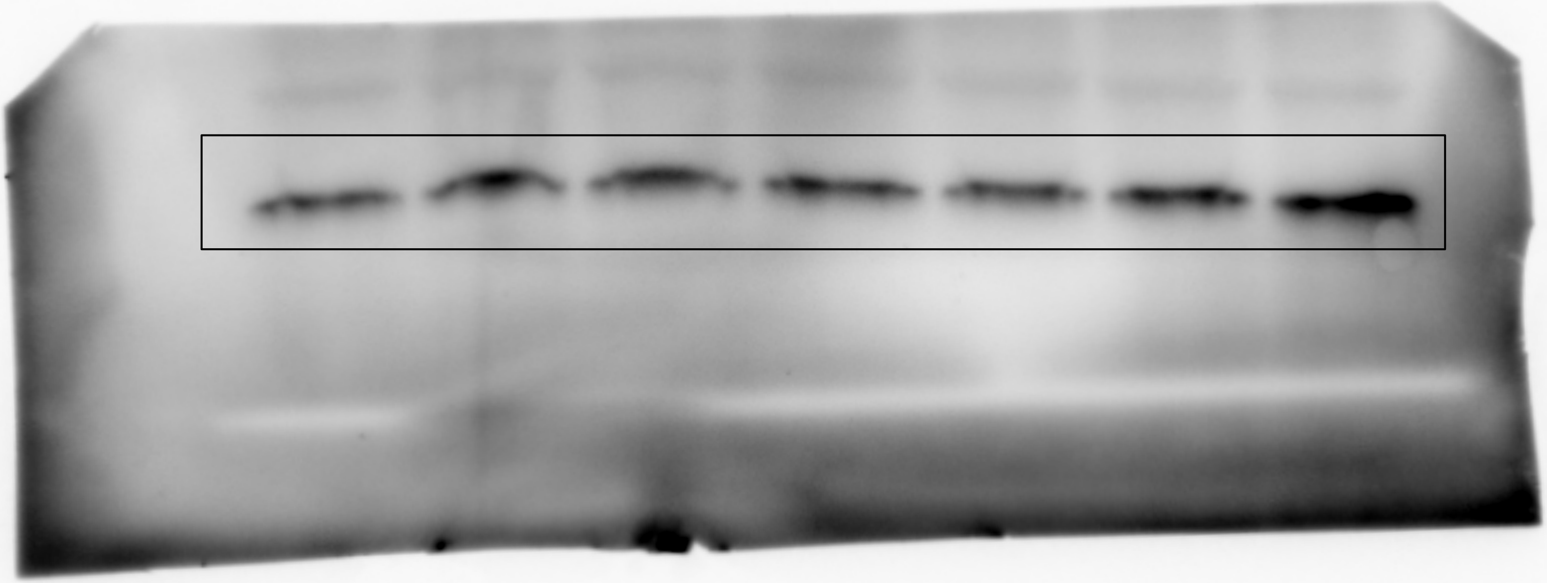

Figure S18

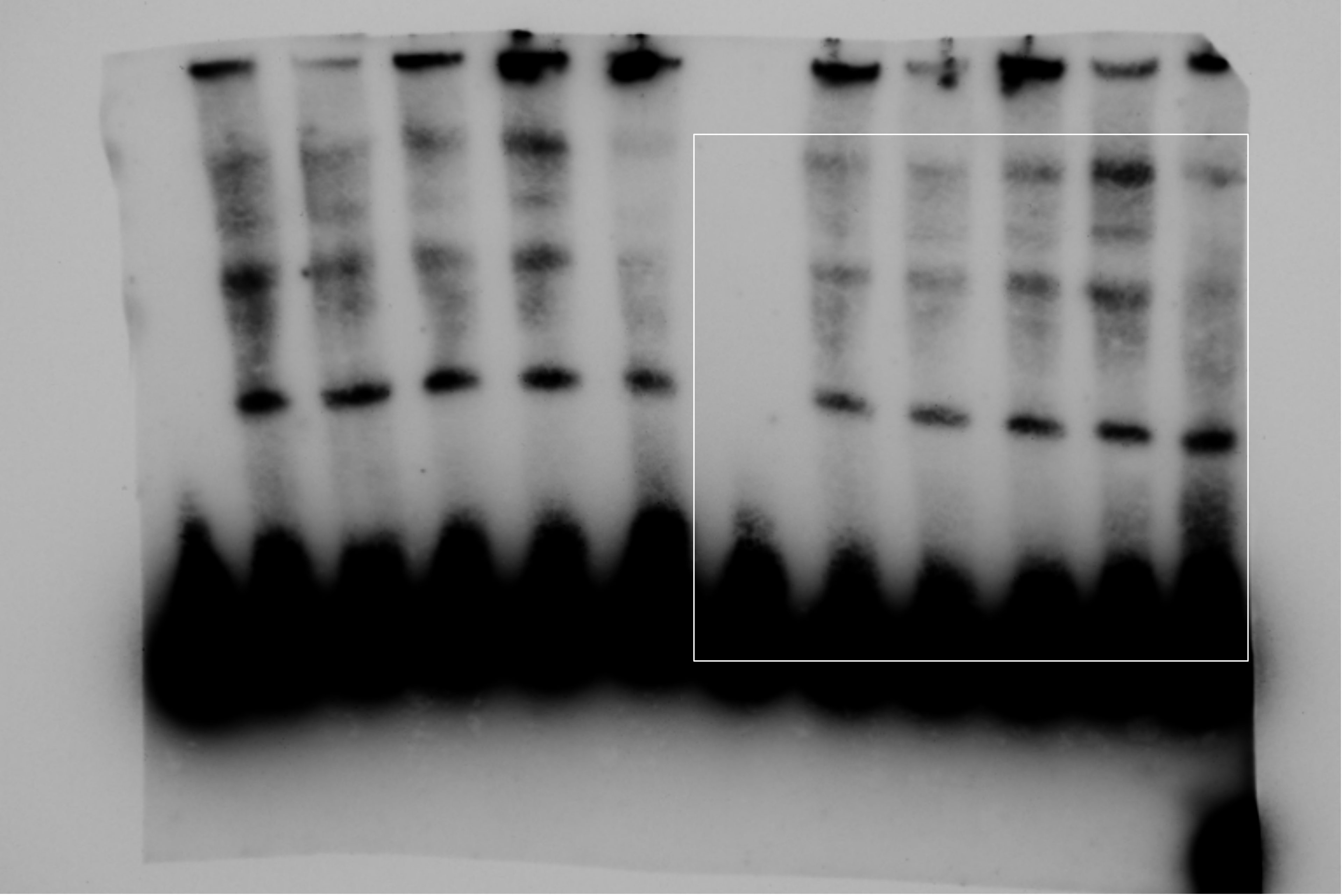

Figure S19

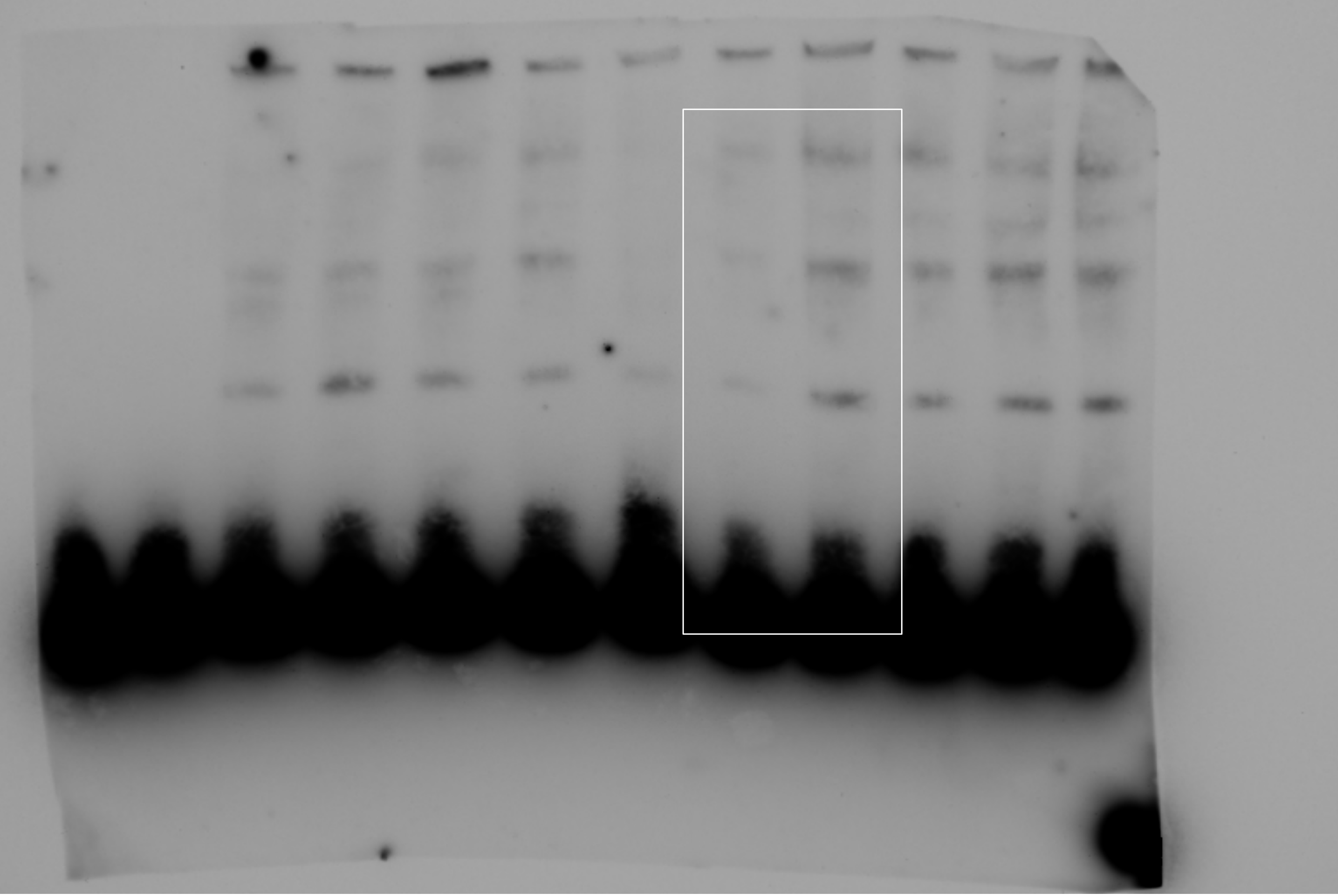

Figure S20

Figure Legends

Supplemental Figure S1

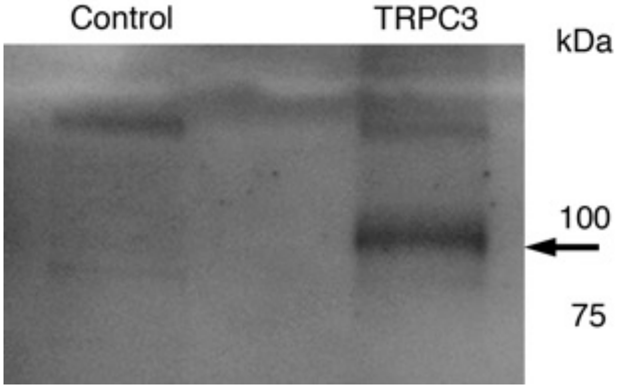

Expression of TRPC3 protein in TRPC3 overexpressing fibroblasts.

The same amount of protein from the lysate of either TRPC3 overexpressing fibroblasts or control empty-vector transfected fibroblasts were loaded on a 10% sodium dodecyl sulfate-polyacrylamide gel and then transferred to a PVDF membrane. An anti-TRPC3 antibody was applied to the membrane, which resulted in robust expression of a 97 kilo dalton protein, corresponding to the size of TRPC3.

Supplemental Figure S2

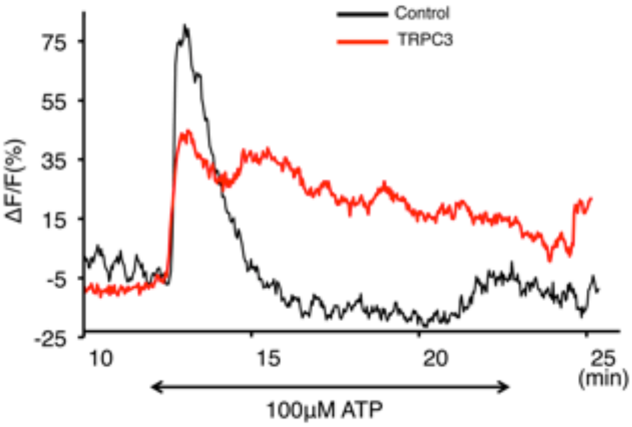

Ca-imaging in TRPC3 overexpressing fibroblasts.

TRPC3 overexpressing fibroblasts and empty-vector transfected control fibroblasts were incubated in 1.5mM Ca modified Krebs solution with 5 μM Fluo-8H and treated with 100 μM ATP. The fluorescent signal of cytoplasmic calcium ion concentration in single fibroblast was determined by fluorescence using confocal laser scanning microscopy (LSM510. Carl Zeiss, Oberkochen, Germany). The fluorescent intensity rose after the treatment in both types of cells. However, the increase of the fluorescent intensity was prolonged in TRPC3 overexpressing fibroblasts compared to the control fibroblasts. X axis: Time observed under microscopy. Y axis: The average of fluorescent intensity of either TRPC3 overexpressing fibroblasts (n=12) or control fibroblasts (n=20).

Supplemental Figure S3

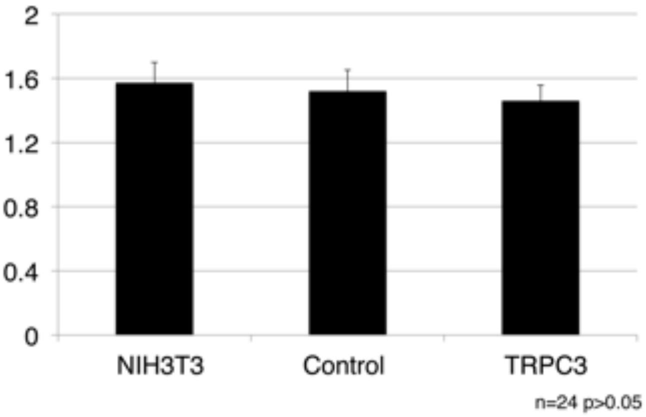

Basal DNA synthesis activity was measured by means of BrdU uptake assay for cell proliferation.

BrdU uptake assay was performed with Cell Proliferation ELISA, BrdU (chemiluminescent) kit (Roche Applied Science Penzberg Germany) according to the manufacturer's protocol. Briefly, either NIH 3T3 fibroblasts, empty-vector transfected control fibroblasts or TRPC3 overexpressing fibroblasts were seeded in DMEM medium supplemented with 10% FBS at a density of 1×10^4/well in a 96 wells microtiter plate and cultured for 24 hour. During the final 2 hour of culture, 10 μM BrdU was added to the wells and BrdU incorporation was then evaluated by measuring the absorbance at 450–540 nm. In regard to proliferation rate, there were no significant differences among these three types of cells (n=24 in each group. p>0.05 by Tukey-Krammer test).

Supplemental Figures S4-20

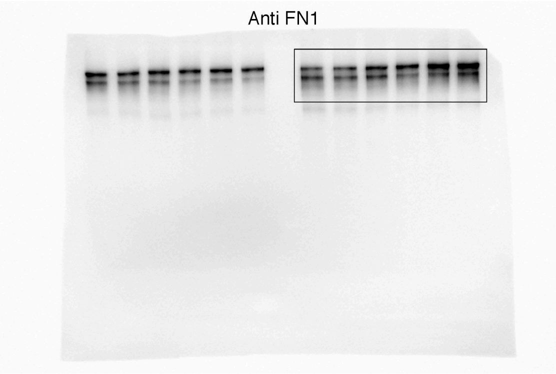

Figure S4

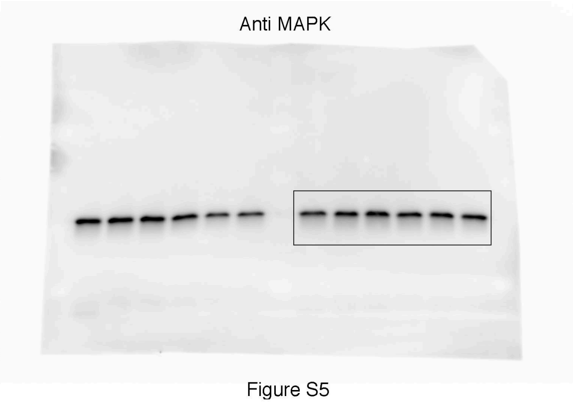

Figure S5

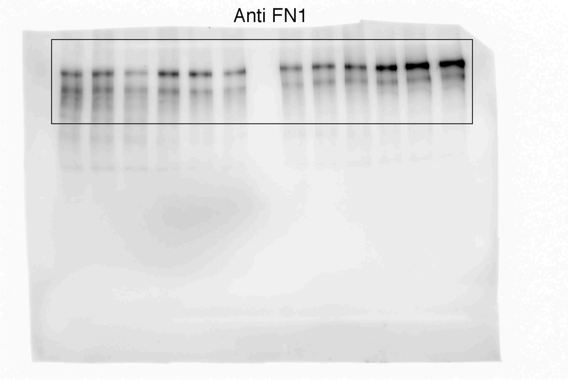

Figure S6

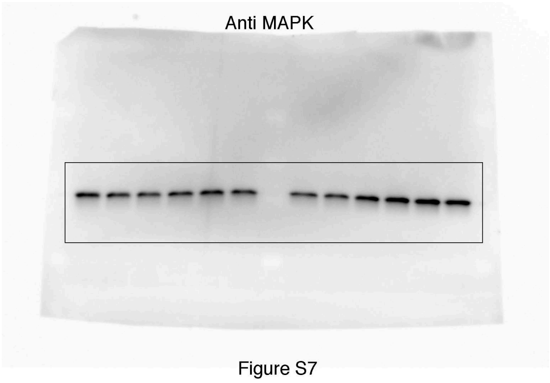

Figure S7

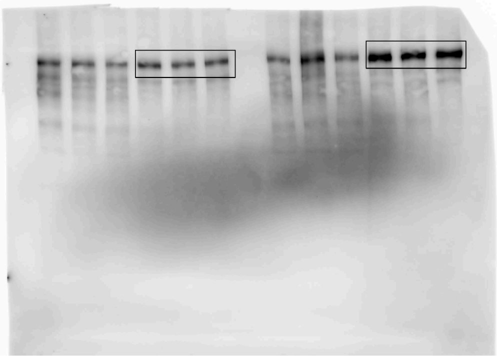

Figure S8

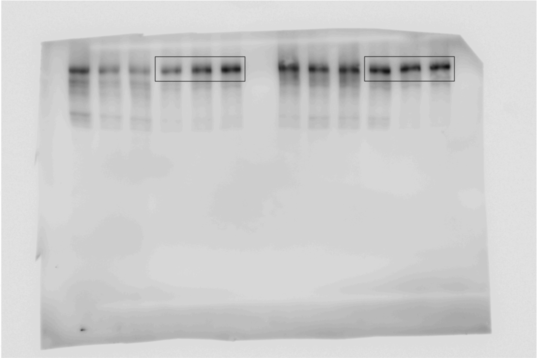

Figure S9

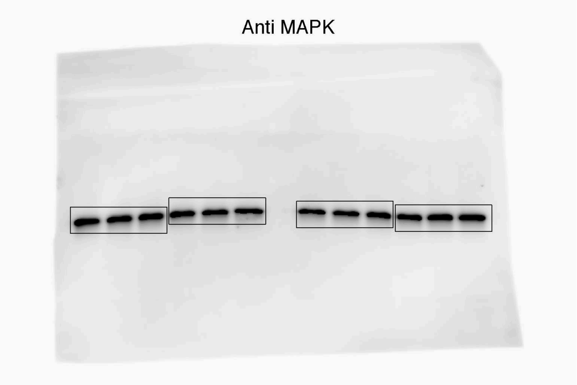

Figure S10

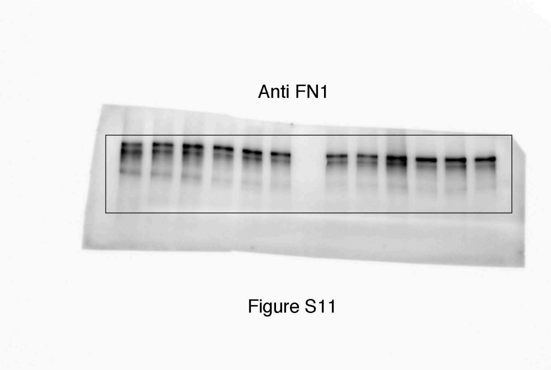

Figure S11

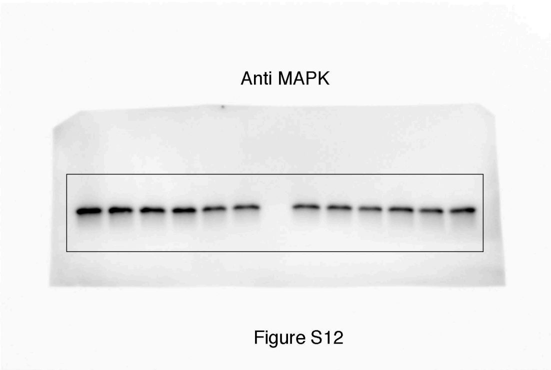

Figure S12

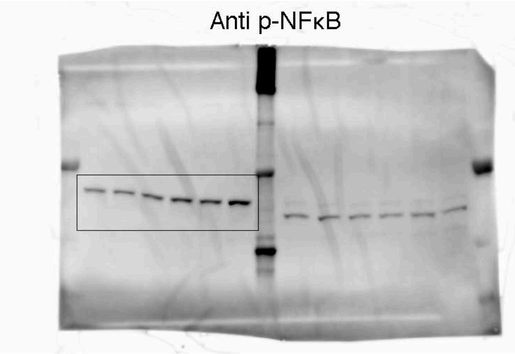

Figure S13

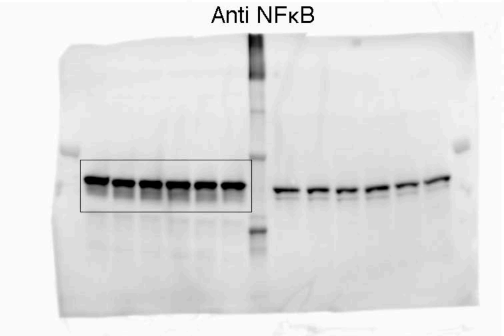

Figure S14

Anti p-NFκB

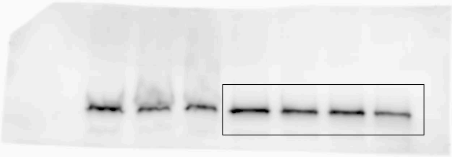

Figure S15

Anti NFκB

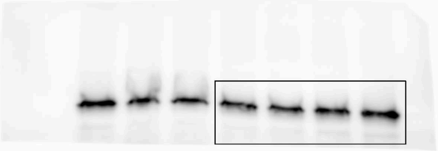

Figure S16

Anti p-IκB

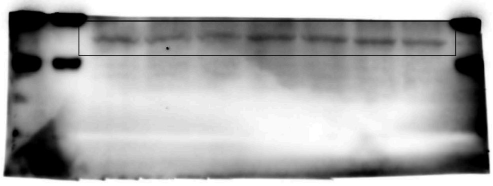

Figure S17

Anti IκB

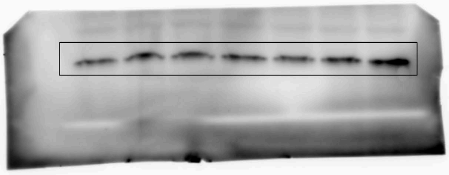

Figure S18

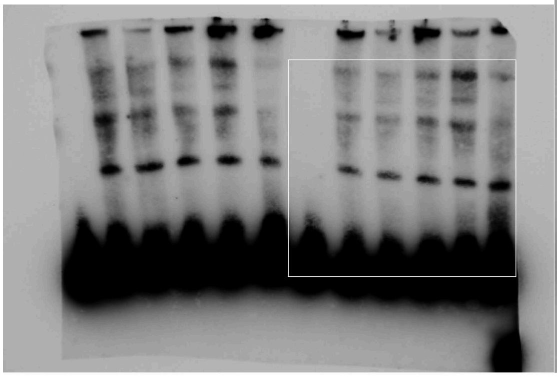

Figure S19

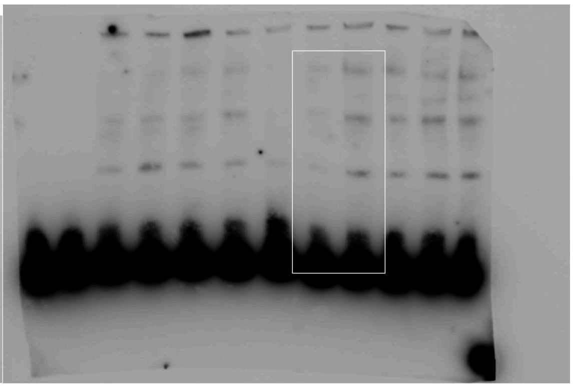

Figure S20

Uncropped, full-length original blots of edited images of figures presented in the manuscript.

The blots were performed with the indicated antibodies. The cropped area was indicated with the solid lines.

Supplemental videos

Ca imaging with a stretch chamber; Real-time calcium influx could be observed while stretching with the remote-controlled single chamber stretcher set on fluorescent microscopy as described in Methods section.

Live video of stretched fibroblasts (Control Cyclic Stretch, TRPC3 Cyclic Stretch1 and 2, and TRPC3 Continuous Stretch); TRPC3 overexpressing fibroblasts showed the rapid increase of fluorescent intensity in response to the repetitive stretching while control cells didn't. TRPC3 overexpressing fibroblasts that were *continuously* stretched experienced decreasing fluorescence over time.
